# Single‐molecule Magnet Properties of Silole‐ and Stannole‐ligated Erbium Cyclo‐octatetraenyl Sandwich Complexes

**DOI:** 10.1002/chem.202500011

**Published:** 2025-02-18

**Authors:** Siddhartha De, Arpan Mondal, Yan‐Cong Chen, Ming‐Liang Tong, Richard A. Layfield

**Affiliations:** ^1^ Department of Chemistry School of Life Sciences University of Sussex Brighton BN1 9QR UK; ^2^ Key Laboratory of Bioinorganic and Synthetic Chemistry of the Ministry of Education School of Chemistry Sun-Yat Sen University Guangzhou 510006 P. R. China

**Keywords:** lanthanides, organometallics, erbium, metallole ligand, single-molecule magnet

## Abstract

The synthesis, structures and magnetic properties of an η^5^‐silole complex and an η^5^‐stannole complex of erbium are reported. The sandwich complex anions [(η^5^‐Cp^Si^)Er(η^8^‐COT)]^–^ and [(η^5^‐Cp^Sn^)Er(η^8^‐COT)]^–^, where Cp^Si^ is [SiC_4_‐2,5‐(SiMe_3_)_2_‐3,4‐Ph_2_]^2–^ (**1_Si_**), Cp^Sn^ is [SnC_4_‐2,5‐(SiMe_3_)_2_‐3,4‐Me_2_]^2–^ (**1_Sn_**) and COT=cyclo‐octatetraenyl, were obtained as their [K(2.2.2‐cryptand)]^+^ salts and found to be isostructural, with remarkably similar bond lengths and angles, differing only in the lengths of the Er–E interactions (E=Si, Sn). The parallels in the molecular structures of **1_Si_** and **1_Sn_** are reflected in their dynamic magnetic properties, which show single‐molecule magnet behaviour in zero applied field, with effective energy barriers of 115±7 and 125±3 cm^–1^, respectively, along with comparable magnetic relaxation times. Analysis of the two complexes using ab initio calculations reveals differences at a quantitative level, but overall similar electronic structures, with the thermally activated relaxation likely to proceed via the first‐excited Kramers doublet. Comparing **1_Si_** and **1_Sn_** with the previously reported germanium analogue **1_Ge_** reveals that swapping one heavier group 14 element for another in complexes of the type [(η^5^‐Cp^E^)Er(η^8^‐COT)]^–^ has a minimal impact on the SMM behaviour.

## Graphical abstract

The synthesis, structures and dynamic magnetic properties of erbium‐silole and erbium‐stannole complexes are reported and compared to those of the analogous erbium‐germole complex. Remarkable similarities in the single‐molecule magnet properties of the three complexes are unearthed, highlighting the minimal role of the heavier group 14 elements in slow magnetic relaxation behaviour.

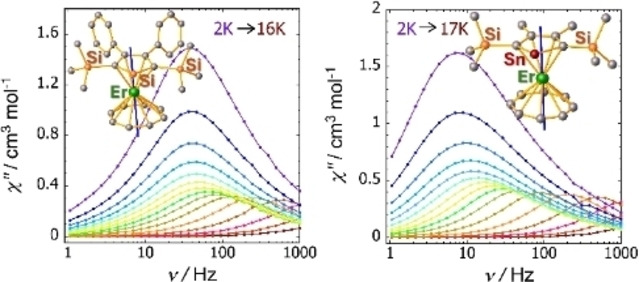

## Introduction

More than two decades have passed since the landmark discovery of slow magnetic relaxation in monometallic terbium and dysprosium phthalocyanine complexes at low temperatures.^[1]^ During this period, an explosion in the number of single‐molecule magnets (SMMs) based on lanthanides has occurred, with the field dominated by dysprosium(III).[^2^, ^3^, ^4^, ^5^] Recent advances highlight a distinct trend in which the key SMM metrics, especially the effective energy barrier to reversal of the magnetization (*U*_eff_) and the magnetic blocking temperature (*T*_B_), have been improved through rational design and innovative synthetic chemistry. For example, symmetry‐based design of dysprosium SMMs has proven to be fruitful for increasing *U*_eff_,[^6^, ^7^, ^8^, ^9^, ^10^, ^11^] as has the use of radical ligands in polynuclear complexes to improve *T*_B_.[^12^, ^13^, ^14^, ^15^, ^16^]

In terms of headline performance parameters, the metallocene blueprint for dysprosium SMMs has produced many eye‐catching results, including successive ‘record‐breaking’ systems with unprecedentedly high energy barriers and blocking temperatures.[^17^, ^18^, ^19^, ^20^, ^21^, ^22^, ^23^] The success of this approach is based largely on a simple consideration of the anisotropic oblate‐shaped 4f^9^ electron density of Dy^3+^, which can be enhanced by placing two negatively charged ligands, such as cyclopentadienyl or [Cp]^–^, ‘above’ and ‘below’ the metal center.[^24^, ^25^, ^26^] Inevitably, more sophisticated theoretical models have revealed that the picture is much more complicated, and a consideration of intramolecular factors alone overlooks the interactions of individual molecules with their environment, especially the phonon bath that plays a large part in activating the relaxation.[^27^, ^28^]

Despite the prevalence of dysprosium metallocene SMMs,[^29^, ^30^, ^31^, ^32^, ^33^] the organometallic approach has also been used to good effect with other lanthanides, particularly erbium. Using a complementary approach, numerous studies have found that ligands with a larger effective radius than cyclopentadienyl provide a match to the anisotropic prolate electron density of the 4f^11^ ion Er^3+^, notably cyclo‐octatetraenyl (COT) and, more recently, cyclo‐nonatetraenyl.[^34^, ^35^, ^36^, ^37^, ^38^, ^39^, ^40^, ^41^, ^42^, ^43^, ^44^, ^45^, ^46^, ^47^] Although the *U*_eff_ and *T*_B_ values determined for SMMs of the type [(η^5^‐Cp^R^)Er(η^8^‐COT)] are typically modest compared to their dysprosium metallocene cousins, these compounds provide an important testbed for relating electronic structure and bonding in erbium sandwich complexes to the dynamic magnetism. Appropriate theoretical modelling of the magnetic properties of some COT‐ligated erbium SMMs may also signpost the use of these materials in quantum computing applications.^[48]^

Our interests in erbium sandwich SMMs focus on the effects of incorporating heavier p‐block elements into the ligand framework. Here, the aim is to understand how, if at all, SMM performance parameters change when a regular cyclopentadienyl ligand is replaced by a metallole ligand of the type [EC_4_R_4_]^2–^ (Cp^E^)[^49^, ^50^] in complexes with the general formula [(η^5^‐Cp^E^)Er(η^8^‐COT)]^–^, where E=Si, Ge, Sn or Pb. Having previously described the SMM properties of germole‐ligated [(η^5^‐Cp^Ge^)Er(η^8^‐COT)]^–^,^[51]^ and discovered that using similar reaction conditions to isolate the silole analogue results in unexpected activation of THF at the silicon(II) centre,^[52]^ we targeted an intact complex of the type [(η^5^‐Cp^Si^)Er(η^8^‐COT)]^–^ using a different synthetic strategy, and a tin version [(η^5^‐Cp^Sn^)Er(η^8^‐COT)]^–^, noting that stannole ligands are currently unknown in f‐element chemistry.

## Results and Discussion

**Scheme 1:**
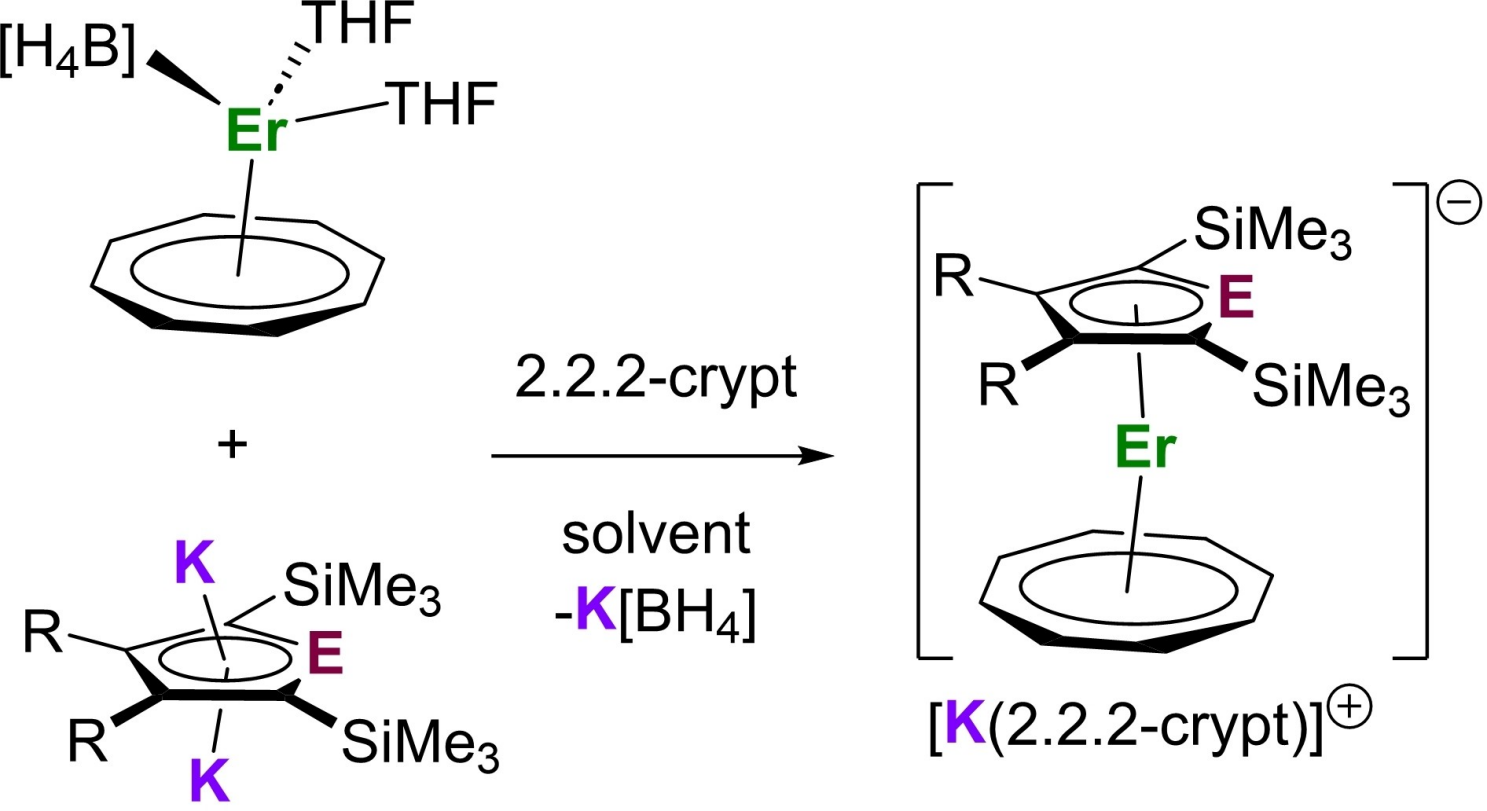
Synthesis of [K(crypt)][**1_Si_**] and [K(crypt)][**1_Sn_**]. E=Si, R=Ph, solvent=1,4‐dioxane; E=Sn, R=Me, solvent=THF.

The target erbium‐silole and erbium‐stannole complexes were synthesized according to Scheme 1. Reasoning that ring strain was responsible for the previously observed oxidative addition of THF across the silicon(II) center of the silole ligand [SiC_4_‐2,5‐(SiMe_3_)_2_‐3,4‐Ph_2_]^2–^, the less‐strained solvent 1,4‐dioxane was used instead. Addition of 1,4‐dioxane to a 1 : 1 : 1 mixture of [(η^8^‐COT)Er(BH_4_)(THF)_2_],^[53]^ the potassium silole [K_2_Cp^Si^⋅1.5(THF)]^[49]^ and 2.2.2‐cryptand (crypt) did indeed generate the desired compound [K(2.2.2‐crypt)][(η^5^‐Cp^Si^)Er(η^8^‐COT)] ([K(crypt)][**1_Si_**]) as red crystals in an isolated yield of 45 %. The erbium‐stannole complex was synthesized using a similar protocol in THF using the potassium stannole K_2_[SnC_4_‐2,5‐(SiMe_3_)_2_‐3,4‐Me_2_] ([K_2_Cp^Sn^⋅2(THF)]), a related lithium stannole being reported previously.^[54]^ The lower nucleophilicity of the tin(II) centre in Cp^Sn^ is evidently tolerant of the ring‐strained THF solvent molecules since no evidence of solvent activation was found. Thus, [K(2.2.2‐crypt)][(η^5^‐Cp^Sn^)Er(η^8^‐COT)] ([K(crypt)][**1_Sn_**]) was isolated as red crystals in 74 % yield.

**Figure 1:**
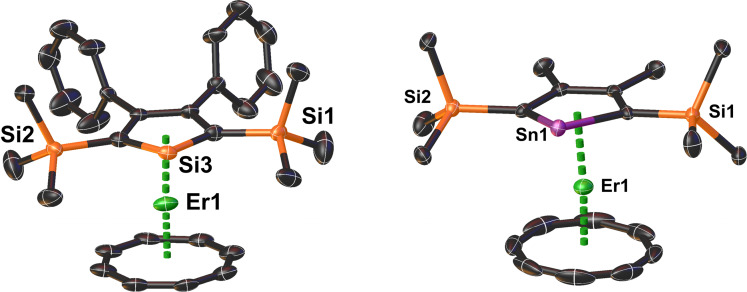
Molecular structures of **1_Si_** (left) and **1_Sn_** (right). Thermal ellipsoids are set to 50 % probability. Unlabelled atoms in black are carbon. For clarity, hydrogen atoms are not shown.

X‐ray crystallography revealed that [K(crypt)][**1_Si_**] and [K(crypt)][**1_Sn_**] are isostructural (Figures 1, Table 1, S1‐S3), which is also reflected in the FTIR spectra of the two compounds (Figures S1‐S3). In both erbium complexes, the metal center is bound by an η^5^‐metallole ligand and an η^8^‐COT ligand, resulting in Cp^E^‐Er‐COT angles of 171.66(15)° and 176.07(7)°, respectively. In **1_Si_**, the Er–C distances to the metallole ligand lie in the range 2.582(7)‐2.609(7) Å (av. 2.597 Å), and in **1_Sn_** the range is 2.595(3)‐2.622(3) Å (av. 2.603 Å). The Er–Si and Er–Sn distances are 2.882(2) Å and 3.0820(4) Å, respectively, consistent with the different radii of the group 14 elements. Despite the differing Er–E distances, the resulting Er–${\mathrm{Cp}}_{\mathrm{cent}}^{E}$ distances are similar at 2.279(3) Å and 2.2758(13) Å for **1_Si_** and **1_Sn_**, respectively (‘cent’ denotes the ring centroid). The range of Er–C distances to the η^8^‐COT ligand in **1_Si_** is 2.471(12)‐2.663(12) Å (av. 2.554 Å), and the range in **1_Sn_** is 2.524(4)‐2.560(4) Å (av. 2.539 Å). The resulting Er–COT_cent_ 1.789(4) and 1.7653(18) Å in **1_Si_** and **1_Sn_**, respectively, i. e., slightly shorter in **1_Sn_**.

**Table 1:** Selected bond lengths (Å) and angles (°) for **1_Si_**, **1_Ge_** and **1_Sn_**.

	1_Si_	1_Ge_^[a]^	1_Sn_
Er–C	2.582(7)‐2.609(7) av. 2.597	2.589(2)‐2.602(2) av. 2.597	2.595(3)‐2.622(3) av. 2.603
Er–E	2.882(2)	2.9152(3)	3.0820(4)
Er–${\mathrm{Cp}}_{\mathrm{cent}}^{E}$	2.279(3)	2.2693(10)	2.2758(13)
Er–C	2.471(12)‐2.663 (12) av. 2.554	2.523(3)‐2.560(3) av. 2.537	2.524(4)‐2.560(4) av. 2.539
Er–COTcent	1.789(4)	1.7610(13) Å	1.7653(18)
COT−Er‐CpE	171.66(15)	175.38(5)	176.07(7)

^[a]^ Data for **1_G_**_e_ are taken from reference 51.^[51]^

Whereas **1_Si_** is only the second example of an intact η^5^‐silole ligand bound to a rare‐earth metal,[^55^, ^56^] **1_Sn_** is seemingly the first example of a rare‐earth stannole complex. Comparing **1_Si_** and **1_Sn_** with the previously reported, isostructural erbium‐germole complex [(η^5^‐Cp^Ge^)Er(η^8^‐COT)]^–^ (**1_Ge_**) reveals their structures to be essentially insensitive to replacement of one heavier group 14 element for another (Table 1).^[51]^ The only noticeable difference between the three structures is the change in Er–E bond length, which proceeds as expected based on the radii of the elements. However, the impact of the lengthening Er–E bonds on the Er–${\mathrm{Cp}}_{\mathrm{cent}}^{E}$ distance is negligible, as it is on the other geometric parameters listed in Table 1. Although the direct plumbole‐ligated version of **1_E_** is not known, Roesky et al. have reported [Li(12‐crown‐4)_2_][(η^5^‐PbC_4_‐2,5‐(Si^*t*^BuMe_2_)_2_‐3,4‐Ph_2_)Er(η^8^‐COT^tips^)] ([Li(12‐crown‐4)_2_][**2_Pb_**]) (COT^tips^=1,4‐*bis*(tri‐isopropylsilyl)cyclooctatetraenyl).[^57^, ^58^] The ligand substituents in **2_Pb_** are clearly much bulkier than those in the lighter congeners, yet the Er–${\mathrm{Cp}}_{\mathrm{cent}}^{\mathrm{Pb}}$ is, at 2.2807(4) Å, similar, as is the Er–COT_cent_ distance of 1.7800(3) Å.

It is instructive to compare the structures of **1_E_** to other, closely related erbium‐COT sandwich complexes. For example, the carbon‐only ‘parent’ complex [(η^5^‐Cp^ttt^)Er(η^8^‐COT)] (Cp^ttt^=1,2,4‐tri(*tert*‐butyl)cyclopentadienyl) has an Er–${\mathrm{Cp}}_{\mathrm{cent}}^{\mathrm{ttt}}$ distance of 2.310 Å and the Er–COT distance is 1.718 Å,^[59]^ i. e., slightly longer (0.03‐0.04 Å) and slightly shorter (0.04‐0.07 Å), respectively, than the equivalent distances in the metallole complexes. The most likely explanation for this difference is that the di‐anionic charge of the [Cp^E^]^2–^ ligands leads to a stronger interaction with the Er^3+^ centre than occurs with [Cp^ttt^]^–^ which, in turn, allows closer approach of the [COT]^2–^ ligand in [(η^5^‐Cp^ttt^)Er(η^8^‐COT)]. The same explanation applies to the structures of phospholyl‐ and arsolyl‐ligated [(η^5^‐Dsp)Er(η^8^‐COT)]^[44]^ (Dsp=3,4‐dimethyl‐2,5‐bis(trimethylsilyl)phospholyl) and [(η^5^‐Dtas)Er(η^8^‐COT)]^[45]^ (Dtas=3,4‐dimethyl‐2,5‐bis(*tert*‐butyl)arsolyl), where the mono‐anionic group 15 hetero‐cyclopentadienyl ligands produce relatively long Er–Dsp_cent_ and Er–Dtas_cent_ distances of 2.321 and 2.3492(3) Å, respectively, and shorter Er–COT distances of 1.686 and 1.7001(2) Å, respectively.

### Magnetic Properties

**Figure 2:**
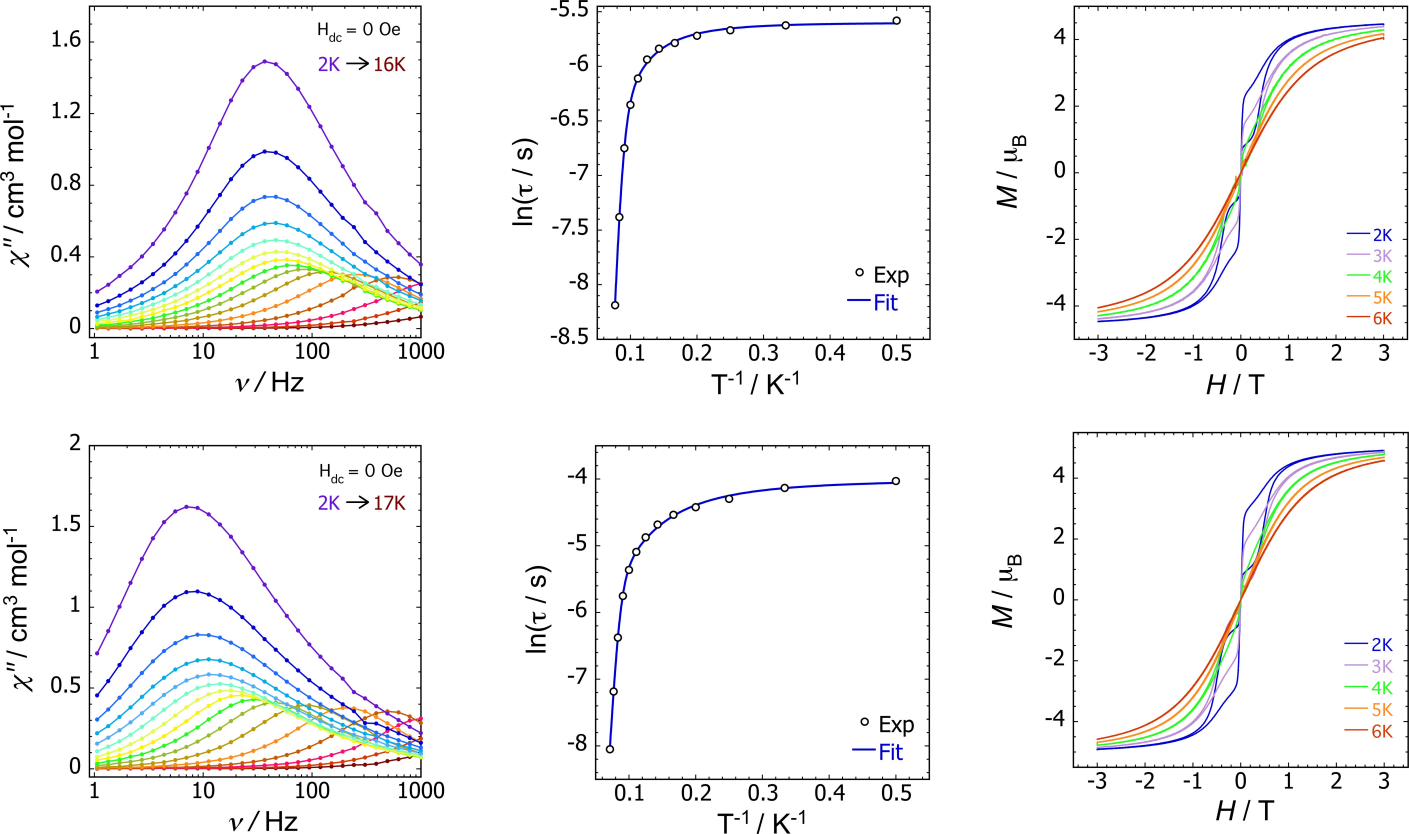
Upper: Plots of 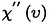 (left), lnτ vs. $T^{-1}$ (centre) and *M*(*H*) hysteresis (right) for [K(crypt)][**1_Si_**]. Lower: equivalent plots for [K(crypt)][**1_Sn_**]. AC susceptibility data were collected in zero DC field at ν= 1–1000 Hz and the temperatures indicated. Relaxation time plots show experimental data points as circles with blue lines representing fits to the data using the parameters stated in Table 2. Hysteresis data were collected using an average field sweep rate of 20 mT s^–1^.

An implication of the structural trends identified for the **1_E_** series is that their magnetic properties, including SMM behaviour, should be similar. Indeed, the structural analysis presented above suggests that the prolate ion Er^3+^ in **1_Si_** and **1_Sn_** will experience a stronger axial crystal field from the [Cp^E^]^2–^ ligands than in [(η^5^‐Cp^ttt^)Er(η^8^‐COT)], [(η^5^‐Dsp)Er(η^8^‐COT)] and [(η^5^‐Dtas)Er(η^8^‐COT)] and, simultaneously, a weaker equatorial crystal field from the [COT]^2–^ ligands, ultimately resulting in lower effective energy barriers for **1_Si_** and **1_Sn_**. To demonstrate the validity of this hypothesis, DC and AC magnetic susceptibility measurements were performed. The DC molar magnetic susceptibility (*χ*_M_) of [K(crypt)][**1_Si_**] and [K(crypt)][**1_Sn_**] was measured in the temperature range 2–300 K using an applied field of *H*_dc_=1000 Oe. The *χ*_M_ *T* value for **1_Si_** is 11.28 cm^3^ K mol^–1^ at 300 K, and for **1_Sn_** it is 11.50 cm^3^ K mol^–1^ (Figures S9, S10), close to the expected value of 11.48 cm^3^ K mol^–1^ for a single Er^3+^ ion with a ^4^I_15/2_ ground term.^[60]^ For [K(crypt)][**1_Si_**], *χ*_M_ *T* decreases gradually with temperature before dropping precipitously around 5 K. For [K(crypt)][**1_Sn_**], *χ*_M_ *T* varies only slightly with decreasing temperature before decreasing below 50 K and then dropping sharply below 5 K. The DC susceptibility for both compounds is consistent with magnetic blocking at low temperatures. In support of this, FC‐ZFC (field‐cooled/zero‐field‐cooled) susceptibility measurements in a field of 1000 Oe for both compounds show a divergence around 5 K (Figures S11, S12). The static field‐dependent magnetization (*M*) was measured at 2–10 K, with saturation values of 4.51 μ_B_ and 5.00 μ_B_ at 2 K and a field of 7 T for [K(crypt)][**1_Si_**] and [K(crypt)][**1_Sn_**], respectively, close to theoretical value of 4.5 μ_B_ for Er^3+^ (Figures S9, S10).^[60]^ Dynamic field‐dependent magnetic hysteresis measurements were also performed using an average field sweep rate of 20 mT s^–1^, with both compounds showing waist‐restricted *M*(*H*) loops at 2 K that remain open to 6 K, broadly consistent with SMM behaviour and the observation of magnetic blocking in the susceptibility measurements (Figure 2).

SMM behaviour for [K(crypt)][**1_Si_**] and [K(crypt)][**1_Sn_**] was confirmed with AC susceptibility measurements in zero DC field and an oscillating (AC) field of *H*_ac_=2 Oe (Figure 2, Figures S13‐S16). The imaginary component of the AC susceptibility (*χ*′′) as a function of frequency (*ν*) reveals similar, although not identical, behaviour. The erbium‐silole complex gives rise to well‐defined maxima in the temperature range 2–13 K and the erbium‐stannole complex shows maxima from 2–14 K (Figure 2). For [K(crypt)][**1_Si_**], the peak values of *χ*′′ are essentially frequency‐independent up to 5 K, whereas for [K(crypt)][**1_Sn_**] a slight dependence of the peak *χ*′′ on frequency occurs at all measured temperatures, becoming more pronounced on warming.

Cole‐Cole plots of the real (*χ*′) versus imaginary component of the susceptibility (Figures S17, S18, Tables S4, S5) were fitted using *α*‐parameters in the range 0.21‐0.06 for the erbium‐silole complex and 0.32‐0.01 for the erbium‐stannole complex, allowing magnetic relaxation times (*τ*) to be extracted. The plot of lnτ vs. $T^{-1}$ for [K(crypt)][**1_Si_**] illustrates the weak temperature dependence of *τ* at low temperatures, indicative of quantum tunnelling of the magnetization (QTM), and a strong temperature dependence at higher temperatures, reflecting thermally activated relaxation. A narrow intermediate temperature regime, with curvature, is also found, where multiple relaxation processes occur simultaneously. Temperature‐independent QTM is less pronounced in [K(crypt)][**1_Sn_**], with lnτ showing a slight dependence on $T^{-1}$ below 5 K, before thermally activated relaxation becomes dominant at higher temperatures.

Good fits of the relaxation time profiles were achieved for both compounds using $\tau^{-1}=\;\tau_{0}^{-1}e^{-U_{eff}/k_{B}T}+CT^{n}+\tau_{QTM}^{-1}$ , where $\tau_{0}^{-1}$ is the attempt rate, *C* is the Raman coefficient, *n* is the Raman exponent, and $\tau_{QTM}^{-1}$ is the QTM rate (Figure 2). As can be seen from the fit parameters in Table 2, the SMM properties of [K(crypt)][**1_Si_**] and [K(crypt)][**1_Sn_**] are indeed very similar, most notably in the case of *U*_eff_, where the two erbium‐metallole complex produce barriers of 115±7 cm^–1^ and 125±3 cm^–1^, respectively. The parameters for the Raman contribution to the relaxation are also very similar, with the relatively low value of *n* being characteristic of organometallic sandwich SMMs.^[61]^ Whilst there is a slight difference in the QTM times for silole and germole complexes, they are a comparable order of magnitude.

**Table 2:** Magnetic relaxation fitting parameters for **1_Si_**, **1_Ge_** and **1_Sn_**.

	1_Si_	1_Ge_^[a]^	1_Sn_
$\tau_{0}$ / s	1.01×10^–9^	9.55×10^–10^	1.14×10^–9^
*U*_eff_ / cm^–1^	115±7	120±1	125±3
*C* / s^–1^ K^–*n*^	0.24±0.11	0.025±0.01	0.57±0.22
*n*	3.0±0.4	3.4±0.4	2.4±0.2
$\tau_{QTM}$ / ms	3.7±0.1	20±4	18±5

^[a]^ Data for **1_Ge_** are taken from reference 51.^[51]^

In comparison with [K(crypt)][**1_Ge_**], the SMM properties of [K(crypt)][**1_Si_**] and [K(crypt)][**1_Sn_**] are also similar. For example, the barrier of 120±1 cm^–1^ determined for **1_Ge_** is intermediate between that of **1_Si_** and **1_Sn_**, although within error the three barriers are essentially equal. The attempt times $\tau_{0}$ and the QTM times of 3.7±0.1 ms, 20±4 ms and 18±5 ms are also comparable. It is also noteworthy that the relaxation times for **1_Ge_** and **1_Sn_** – which have the same substituents and differ only in the heavier group 14 element – are very closely matched at low temperatures. Thus, at 2–14 K, *τ*=18.9‐0.2 ms for **1_Ge_**,^[51]^ and *τ*=17.8‐0.3 ms for **1_Sn_**. Aside from the Raman coefficient for the germole‐ligated complex, the high‐ and low‐temperature magnetic relaxation regimes of the three metallole‐ligated erbium‐COT complexes vary only slightly upon changing the heavier group 14 element, suggesting that the parallels in their molecular structures give a reliable qualitative prediction of their SMM performance. Furthermore, the *U*_eff_ values determined for **1_E_** are indeed markedly lower than those found for [(η^5^‐Cp^ttt^)Er(η^8^‐COT)] (228 cm^–1^),^[59]^ [(η^5^‐Dsp)Er(η^8^‐COT)] (249 cm^–1^)^[44]^ and [(η^5^‐Dtas)Er(η^8^‐COT)] (225 cm^–1^),^[45]^ in line with the proposed magneto‐structural correlation.

It is also interesting to compare the SMM parameters of **1_E_** with those of **2_Pb_**, which shows *U*_eff_=42 cm^–1^ and $\tau_{QTM}=$ 0.25 ms.^[57]^ If the magneto‐structural correlation identified for **1_E_** is valid, the very similar molecular structure of **2_Pb_** should result in similar SMM properties, but this is evidently not so. A possible explanation for the discrepancy is that the secondary coordination sphere in the plumbole‐ligated complex, i. e., the bulky COT substituents, play a role in diminishing the SMM properties. If correct, this would be consistent with observations on sandwich complexes of the type [M(C_8_H_6_‐1,*n*‐R_2_)_2_]^–^ and [M_2_(C_8_H_6_‐1,*n*‐R_2_)_3_] (M=Dy, Er), where R=SiMe_3_ or SiMe_2_^*t*^Bu and *n*=1‐3, where the substituents are thought to skew the easy axis of magnetization away from the molecular symmetry axis, dramatically impacting on the magnetic relaxation times.[^46^, ^62^, ^63^, ^64^, ^65^, ^66^, ^67^] In contrast, use of the parent [COT]^2–^ ligand in **1_E_** is more beneficial for the magnetic axiality, an idea that we sought to explore with the aid of ab initio calculations.

### Theoretical Studies

Multireference CASSCF/QDPT/SINGLE ANISO calculations were performed on **1_Si_** and **1_Sn_** using the ORCA software package,^[68]^ which yielded the *g*‐tensors, a visualization of the easy axes of magnetization, and the crystal field parameters (Tables S6‐S9) of the low‐lying excited state using previously calculated spin‐orbit states.[^69^, ^70^, ^71^, ^72^, ^73^, ^74^, ^75^]

**Figure 3:**
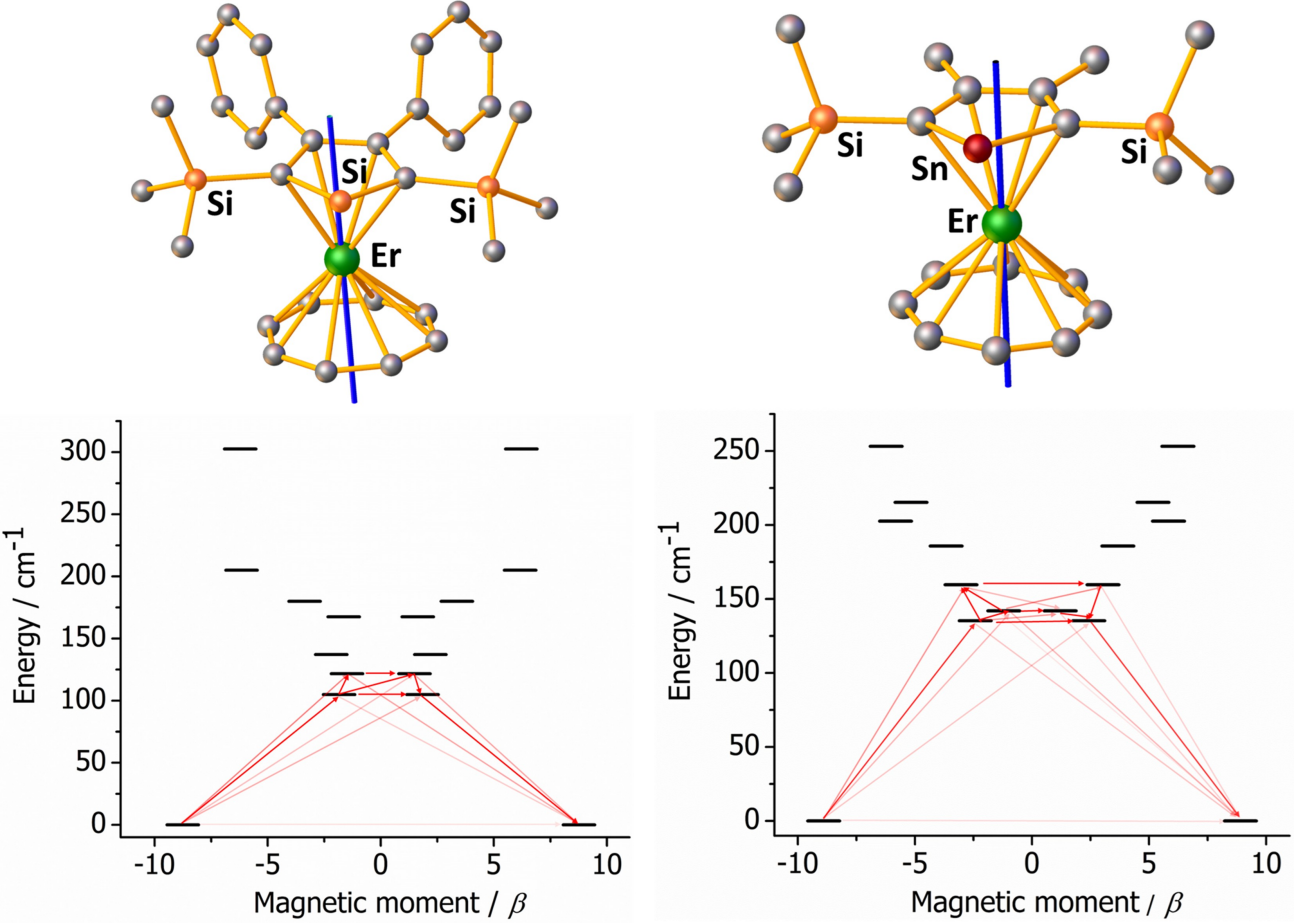
Upper: calculated easy axis of magnetization (blue line) in the ground KD for **1_Si_** (left) and **1_Sn_** (right). For clarity, hydrogen atoms are omitted. Lower: Barrier‐like relaxation for **1_Si_** (left) and **1_Sn_** (right). Darker shading of the red arrows indicates larger absolute value of the transition magnetic moment matrix elements between the respective states. Transitions involving higher‐energy states not involved in the relaxation mechanism are omitted for clarity.

Although quantitatively different, the electronic structures of the silole‐ and stannole‐ligated complexes are qualitatively alike, showing total crystal field splittings across the eight Kramers Doublets (KDs) of 303 cm^–1^ and 253 cm^–1^, respectively. Both complexes have highly axial ground KDs, with *g_x_* =0.0075, *g_y_* =0.0114 and *g_z_* =17.52 for **1_Si_** and *g_x_* =0.0003, *g_y_* =0.0012 and *g_z_* =17.80 for **1_Sn_**. The ground KDs are effectively composed of a single wavefunction corresponding to |*M_J_* |=15/2. The corresponding easy axis of magnetization in **1_Si_** passes close to the center of the Cp^Si^ and COT rings but with a slight skewing of 5.0°, with a similar picture for **1_Sn_** and a skew angle of 4.2° (Figure 3). In contrast, the *g*‐tensors in the first‐excited KDs feature non‐negligible transverse components and are represented by strong admixtures of wavefunctions. Based on the calculations, the thermally activated magnetic relaxation in **1_Si_** and **1_Sn_** should therefore proceed via a barrier‐crossing transition from the first‐excited KD either to the opposite component of the first excited KD or to the second‐excited KD, which lies only slightly higher in energy in both complexes. Assuming barrier‐crossing within the first‐excited KD is dominant (based on the calculated *g*‐tensors), the predicted barriers of 105 cm^–1^ and 133 cm^–1^ in **1_Si_** and **1_Sn_**, respectively, agree well with the barriers extracted from the experimental data (Figure 3, Tables S10, S11). Notably, the magnetic relaxation in **1_Ge_** is also thought to proceed via the first‐excited KD. Thus, in addition to the consistency between the bulk magnetic properties of the silole, germole and stannole complexes **1_E_**, their behaviour at a microscopic level is essentially the same, further emphasizing the limited role played by the heavier group 14 element in erbium SMMs of this type.

## Conclusions

The erbium‐COT sandwich complex **1_Si_** is a rare example of an η^5^‐silole ligand in lanthanide chemistry, and the η^5^‐stannole analogue **1_Sn_** appears to be the first lanthanide complex of this tin‐containing heterocycle. The only appreciable difference in the molecular structures of the two complexes is the Er–E distances of 2.882(2) Å and 3.0820(4) Å, respectively, with the Er–${\mathrm{Cp}}_{\mathrm{cent}}^{\mathrm{Si}}$ and Er–${\mathrm{Cp}}_{\mathrm{cent}}^{\mathrm{Sn}}$ distances being essentially the same, and the Er–COT distances differing by only approximately 0.02 Å. The isostructural nature of the two complexes is projected onto their dynamic magnetic properties, with both showing SMM behaviour according to bulk FC‐ZFC magnetic susceptibility and magnetization measurements. The AC susceptibility responses of silole‐ and stannole‐ligated erbium‐COT complexes up to around 14 K reflect a combination of quantum tunnelling relaxation at low temperatures, Orbach‐type relaxation at higher temperatures, with Raman relaxation at intermediate temperatures. The similar *U*_eff_ values of 115±7 cm^–1^ for the silole complex and 125±3 cm^–1^ for the stannole complex are consistent with their isostructural nature, and with the SMM parameters determined for the germole‐ligated analogue **1_Ge_**. Ab initio calculations reveal further coincidences in the electronic structure at a microscopic level, with dominant thermal relaxation via the first‐excited Kramers doublet in both systems. Our results suggest that, at least for erbium SMMs of the type [(η^5^‐Cp^E^)Er(η^8^‐COT)]^–^ (E=Si, Ge, Sn), the effects of varying the heavier group 14 element on the dynamic magnetic properties and electronic structure should be minimal, provided the secondary coordination sphere remains constant.

## Supporting Information

Synthesis, spectroscopic characterization, crystallography details, magnetic measurements, and computational details can be found in the Supporting Information. The authors have cited additional references within the Supporting Information. Data that support the findings of this study are openly available at DOI: 10.25377/sussex.28123121. Deposition Number(s) 2386821 (**1**_**Si**_), 2386818 (K_2_Cp^Sn^), and 2386820 (**1**_**Sn**_) contain(s) the supplementary crystallographic data for this paper. These data are provided free of charge by the joint Cambridge Crystallographic Data Centre and Fachinformationszentrum Karlsruhe Access Structures service.

## Conflict of Interests

The authors declare no conflict of interest.

1

## Supporting information

As a service to our authors and readers, this journal provides supporting information supplied by the authors. Such materials are peer reviewed and may be re‐organized for online delivery, but are not copy‐edited or typeset. Technical support issues arising from supporting information (other than missing files) should be addressed to the authors.

Supporting Information

## Data Availability

The data that support the findings of this study are openly available in Figshare at https://doi.org/10.25377/sussex.28123121, reference number 1.
