# Supplementary material for: Single‐molecule Magnet Properties of Silole‐ and Stannole‐ligated Erbium Cyclo‐octatetraenyl Sandwich Complexes
Source: Chemistry. 2025 Feb 18;31(17):e202500011. doi: 10.1002/chem.202500011 (PMC11924996; doi:10.1002/chem.202500011)
Supplement: Supplementary file 1 — Supporting Information [file CHEM-31-e202500011-s001.pdf]

Chemistry–A European Journal

Supporting Information

Single-molecule Magnet Properties of Silole- and Stannole-ligated Erbium Cyclo-octatetraenyl Sandwich Complexes

Siddhartha De, Arpan Mondal, Yan-Cong Chen, Ming-Liang Tong,* and Richard A. Layfield*

Supporting Information

Single-molecule Magnet Properties of Silole- and Stannole-ligated Erbium Cyclo-octatetraenyl Sandwich Complexes

Siddhartha De, Arpan Mondal, Yan-Cong Chen, Ming-Liang Tong,* Richard A. Layfield*

Department of Chemistry, School of Life Sciences, University of Sussex, Brighton, BN1 9QR, UK.
E-mail: r.layfield@sussex.ac.uk

Key Laboratory of Bioinorganic and Synthetic Chemistry of the Ministry of Education, School of Chemistry, Sun-Yat Sen University, Guangzhou 510006, P. R. China.
E-mail: tongml@mail.sysu.edu.cn

Contents

Synthesis Details S1-S2
FTIR Spectra S2-S3
NMR Spectra S4-S5
X-ray Crystallography S6-S9
Magnetic Property Measurements S10-S15
Computational Details S16-S21
References S22

Synthesis Details

General Considerations

All synthetic experiments were carried out under anhydrous and anaerobic conditions using standard Schlenk techniques and argon-filled MBraun gloveboxes. Solvents were refluxed over a suitable drying agent for a minimum of three days (molten potassium for THF and 1,4-dioxane, and Na/K alloy for hexane), and then distilled, degassed via a minimum of three freeze-pump-thaw cycles, and stored in ampoules over potassium mirrors (hexane) and activated 4 Å molecular sieves (THF and 1,4-dioxane). Literature procedures were used to prepare the potassium silole [K_2Cp^{Si}·1.5THF],[48] 1,1-dichloro-2,5-trimethylsilyl-3,4-dimethylstannole,[53] and [Er(COT)(BH_4)($THF)_2$].[52] 2.2.2-Cryptand was purchased from Sigma-Aldrich and dried under vacuum at 60 °C for three days before use. All other chemicals were obtained from commercial sources and used without further purification.

All NMR spectra were recorded in THF-D_8, which was dried over potassium and degassed by freeze-pump-thaw cycles. NMR spectra were recorded on a Varian VNMR S400 spectrometer operating at 30°C at frequencies of 400 MHz (^{1}H), 80 MHz (^{29}Si), 100 MHz (^{13}C), and 149 MHz (^{119}Sn). The ^{1}H and ^{13}C chemical shifts were calibrated to the residual signals from the deuterated solvent. Multiplicity of the signals is indicated as s = singlet, d = doublet, dd = doublet of doublets, t = triplet, q = quartet and m = multiplet. NMR samples were prepared inside the glovebox using NMR tubes fitted with J. Young valves.

Elemental analyses were performed at Elemental Microanalysis Limited, Devon, United Kingdom. Attenuated total reflectance Fourier-transform infrared spectroscopy (ATR-FTIR) spectra were collected using a Bruker ALPHA spectrometer equipped with a Platinum ATR module in a glovebox.

Synthesis of [K(2.2.2-crypt)][(η^8-COT)Er(η^5-Cp^{Si}] (1_{Si})

Dioxane (15 ml) was added to a mixture of [K_2Cp^{Si}·1.5THF] (56 mg, 0.1 mmol), [Er(COT)(BH_4)($THF)_2$] (45 mg, 0.1 mmol) and 2.2.2-cryptand (38 mg, 0.1 mmol) at room temperature. After stirring for 30 minutes, THF (5 ml) was added to the mixture and the mixture was stir for 20 hours at room temperature. The resultant orange-red suspension was allowed to settle for 10 minutes and filtered. The filtrate was evaporated to

dryness and extracted with THF. The THF-soluble fraction was layered with hexane at room temperature. Red crystals were isolated after six days (48 mg, 45%). **Elemental analysis** calculated for $C_{48}H_{72}ErKN_2O_6Si_3$: C, 54.2; H, 6.82; N, 2.63. Found: C, 54.84; H, 6.91; N, 2.66.

Synthesis of [K_2Cp^{Sn}·2(THF)]

THF (50 ml) was added to a mixture containing 1,1-dichloro-2,5-trimethylsilyl-3,4-dimethylstannole (829 mg, 2 mmol) and freshly cut potassium (783 mg, 20 mmol) at room temperature. The colour of the solution became green after 15 minutes and the mixture was allowed to stir at room temperature for 72 hours. The resulting dark yellow solution was filtered and evaporated to dryness. The resulting solid was redissolved in THF, filtered, and the THF-soluble fraction was layered with hexane and stored at –20 °C. Yellow crystals were isolated after two days (938 mg, 83%,). **Elemental analysis** calculated for $C_{20}H_{40}SnSi_2K_2O_2$: C, 42.47; H, 7.13. Found: C, 42.15; H, 7.37. **^{1}H NMR** (δ/ppm): 0.19 (18H, s, SiMe), 2.28 (6H, s, CH_3, Cp^{Sn}). **^{13}C{^{1}H} NMR** (δ/ppm): 6.17 ($SiMe_3$), 26.56 (CH_3, Cp^{Sn}), 136.23 (C_β, Cp^{Sn}), 175.22(C_α, Cp^{Sn}). **^{119}Sn{^{1}H} NMR** (δ/ppm): 433.9. **^{29}Si{^{1}H} NMR** (δ/ppm): –15.34.

Synthesis of [K(2.2.2-crypt)][(η^8-COT)Er(η^5-Cp^{Sn}] (1_{Sn})

THF (10 ml) was added to a mixture containing [K_2Cp^{Sn}·2THF] (56 mg, 0.1 mmol), [Er(COT)(BH_4)$(THF)_2$] (45 mg, 0.1 mmol) and 2.2.2-cryptand (38 mg, 0.1 mmol) at room temperature. The dark red mixture was mixture was allowed to stir for 20 hours at room temperature. The resultant red suspension was filtered. The filtrate was evaporated to dryness and extracted with THF. The THF-soluble fraction was layered with hexane at room temperature. Red crystals were isolated after three days (76 mg, 74%). Elemental analysis calculated for $C_{38}H_{68}ErKN_2O_6Si_2Sn$: C, 44.3; H, 6.65; N, 2.72. Found: C, 44.84; H, 6.87; N, 2.82.

FTIR Spectra

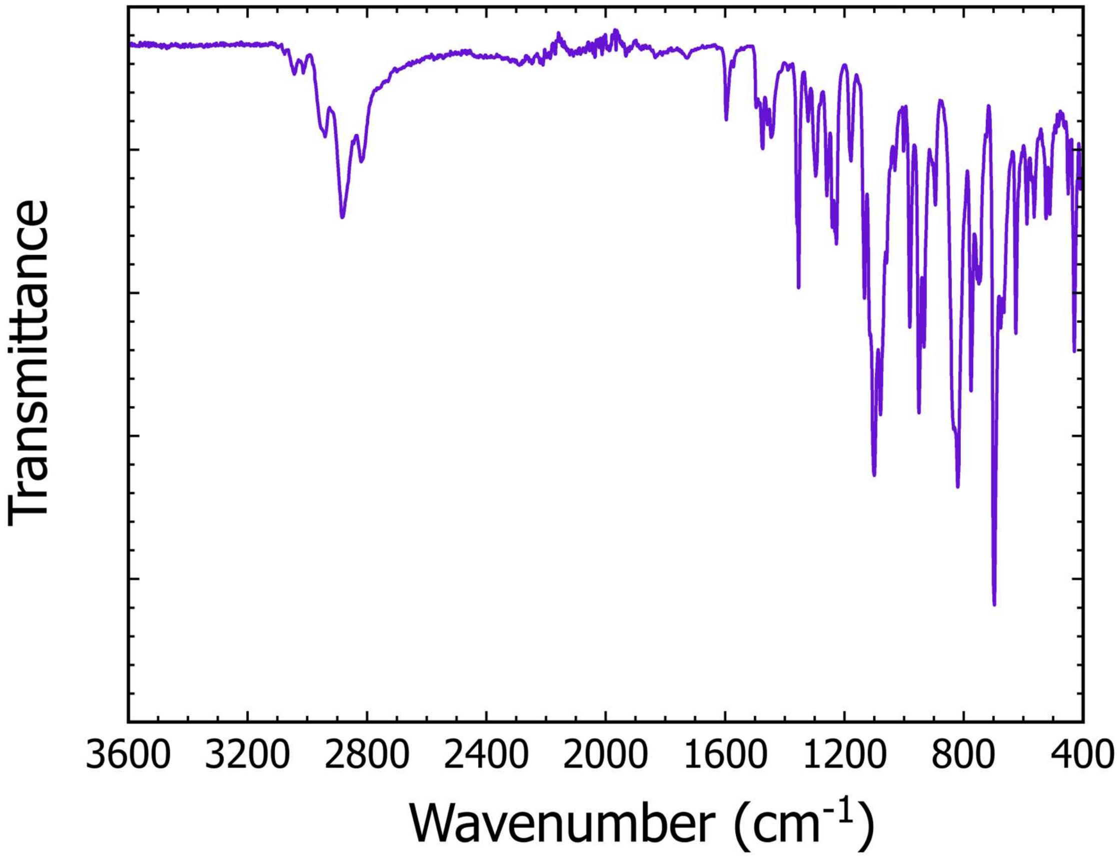

Figure S1. Solid-state FTIR spectrum of 1_{Si}.

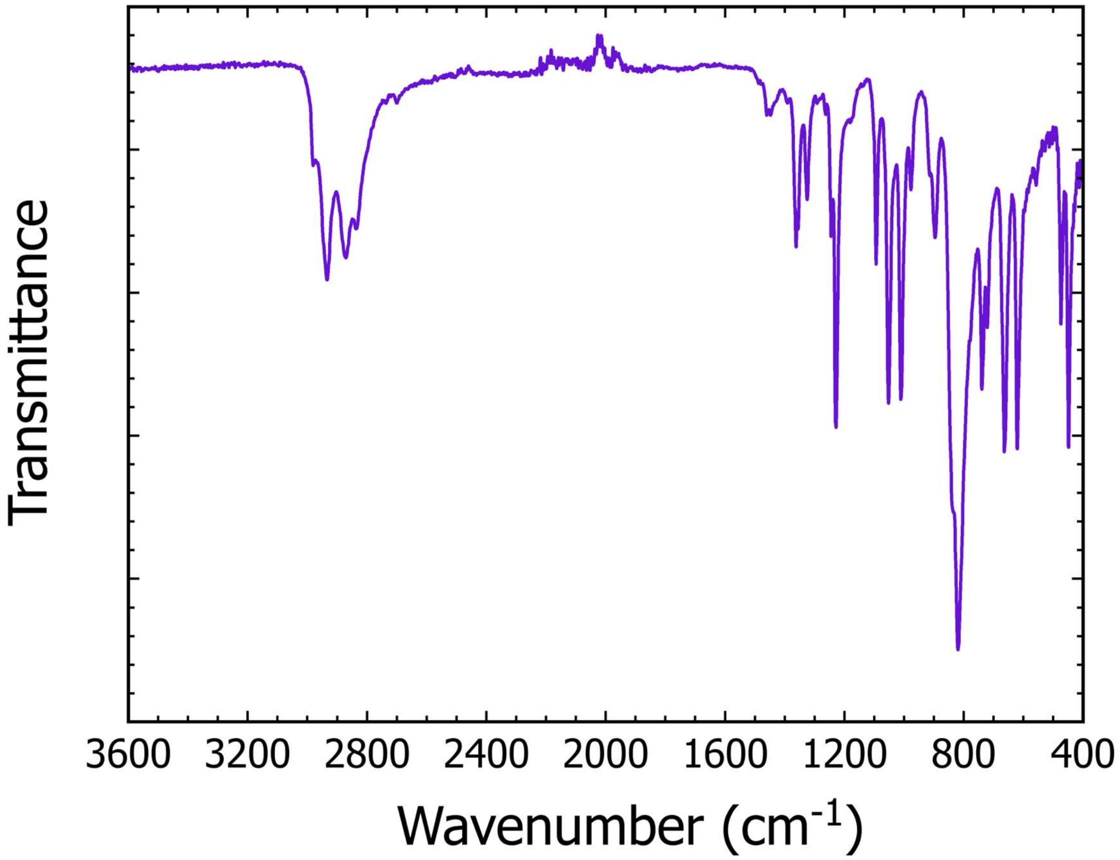

Figure S2. Solid-state FTIR spectrum of K_2Cp^{Sn}·2THF.

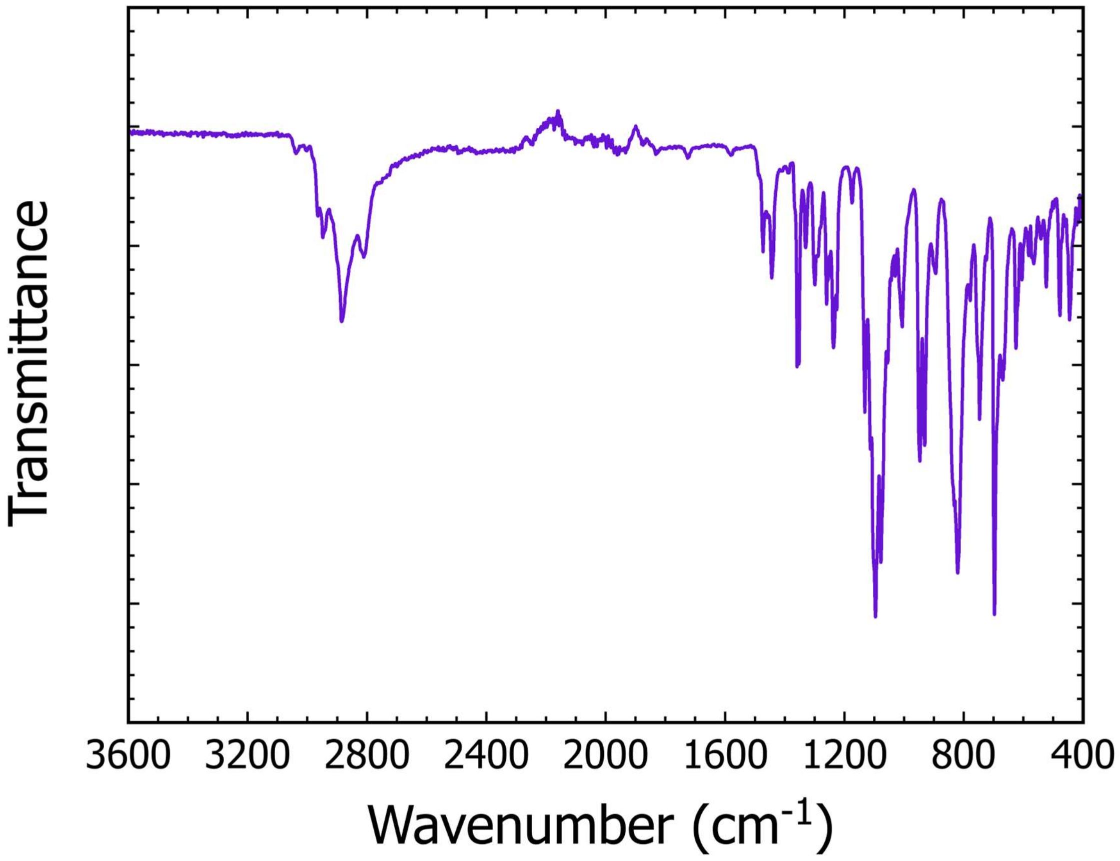

Figure S3. Solid-state FTIR spectrum of $\mathbf{1_{Sn}}$.

NMR Spectra

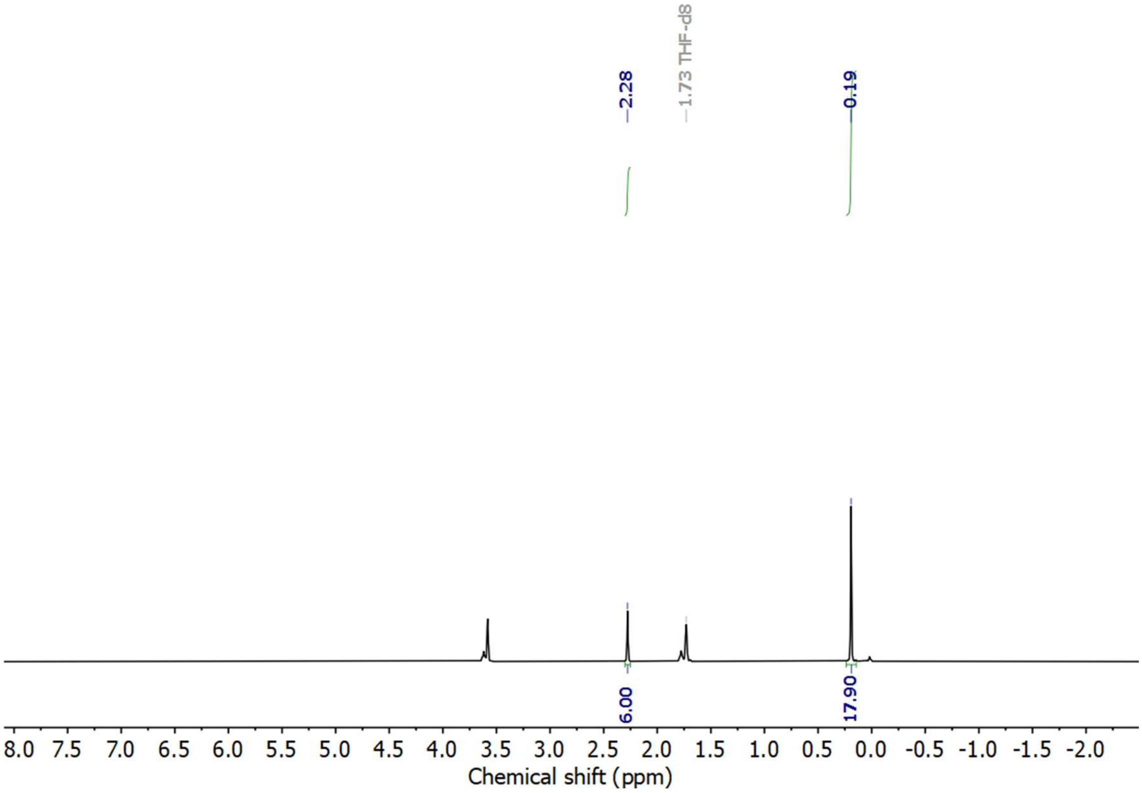

Figure S4. ^{1}H NMR spectrum of $K_2Cp^{Sn}·2THF$ in THF-D_8.

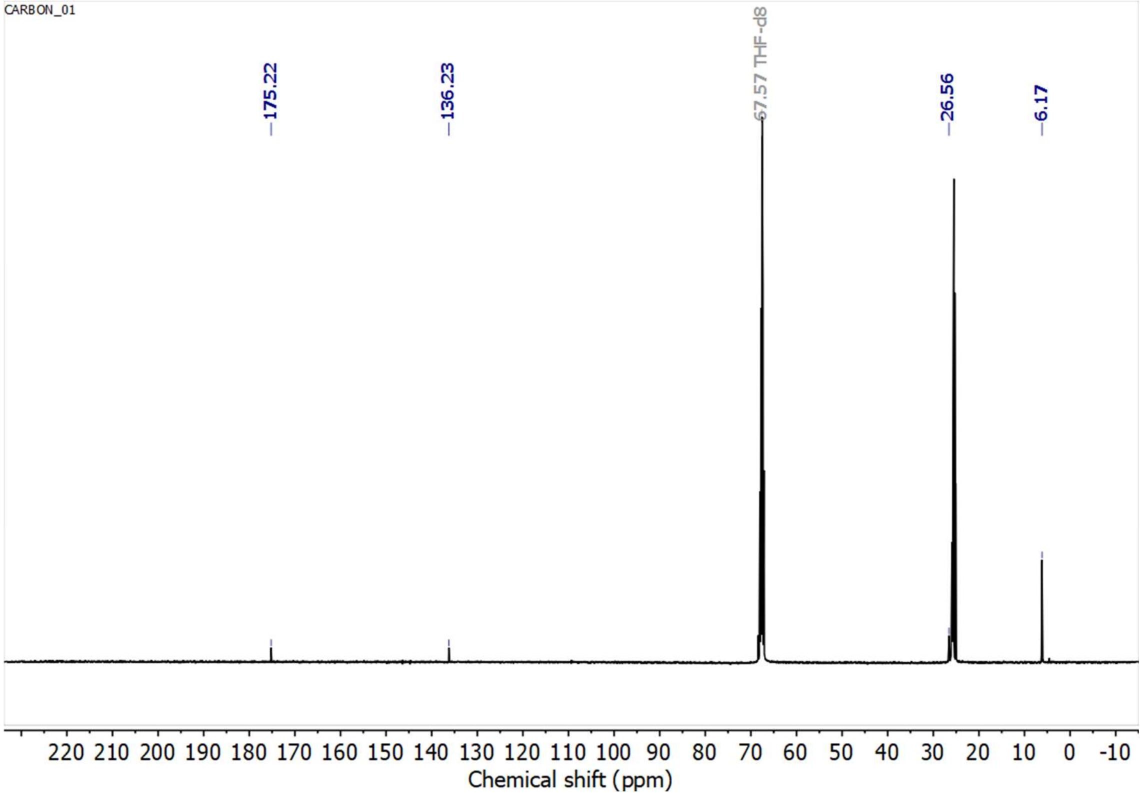

Figure S5. $^{13}C\{^{1}H\}$ NMR spectrum of $K_2Cp^{Sn}·2THF$ in THF-D_8.

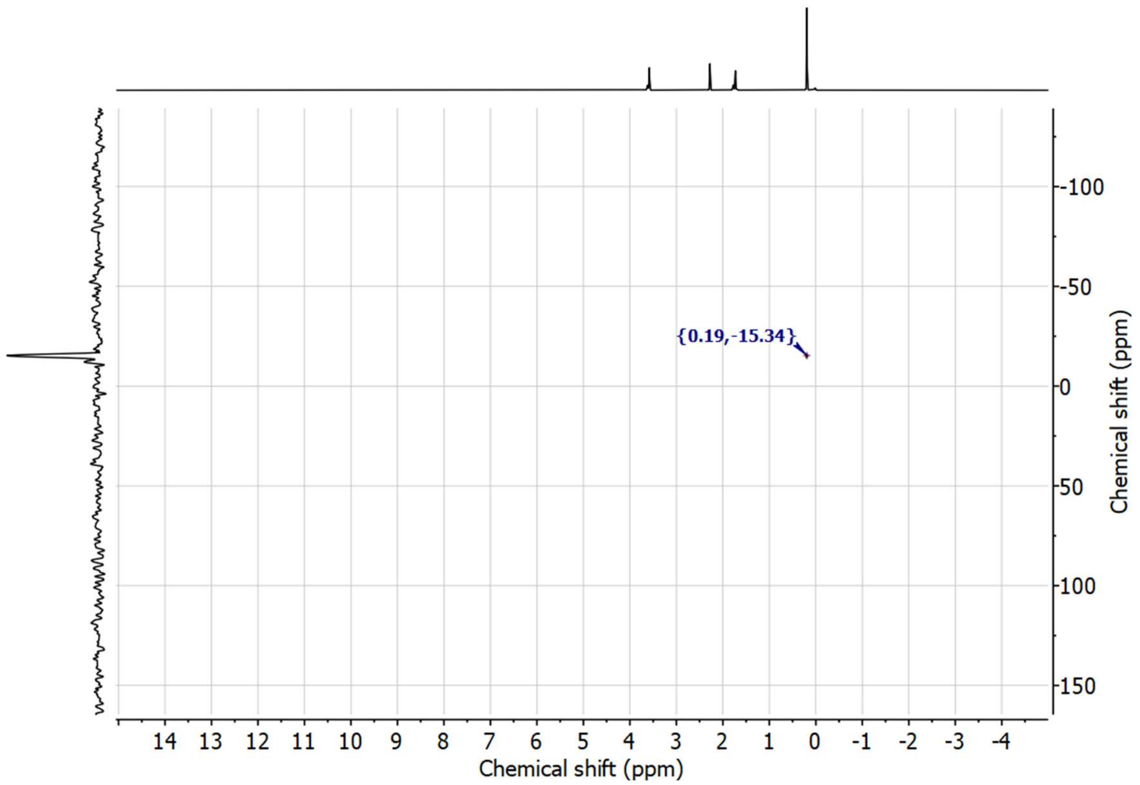

Figure S6. $^{1}H/^{29}Si$-HMBC-NMR spectrum of $K_2Cp^{Sn}\cdot 2THF$ in THF-D_8**.**

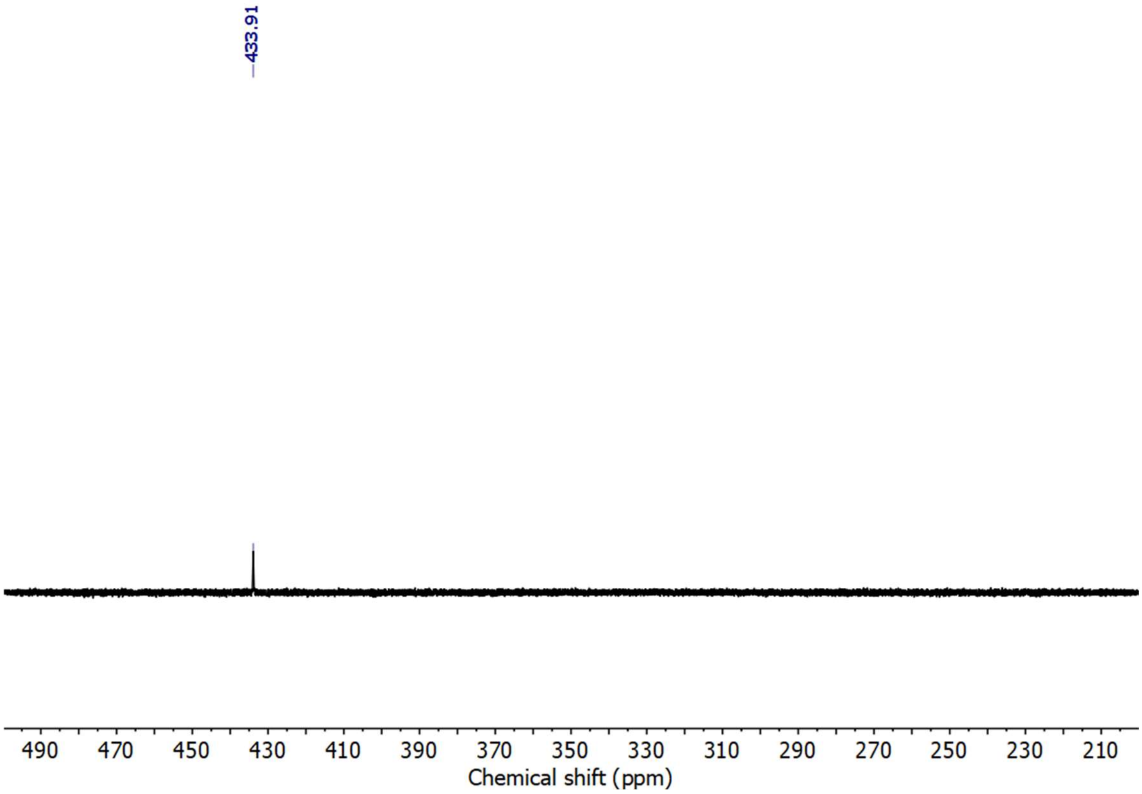

Figure S7. $^{119}Sn\{^{1}H\}$ NMR spectrum of $K_2Cp^{Sn}\cdot 2THF$ in THF-D_8.

X-ray crystallography

Single crystals were immersed in NVH oil (degassed and dried) and mounted on a Bruker D8 Venture Metaljet diffractometer. Ga-K_{α} radiation (λ = 1.34139 Å) was used in each case. Using Olex2,[70] structures were solved with the olex2.solve structure solution program using charge flipping[71] and refined with the SHELXL refinement package using least squares minimization.[72] Crystallographic data were deposited at Cambridge Crystallographic Data Centre and are available via https://www.ccdc.cam.ac.uk/structures/.

Table S1. Crystal data for K_2Cp^{Sn}·2(THF), $\mathbf{1}_{Si}$ and $\mathbf{1}_{Sn}$.

	$\mathbf{1}_{Si}$	$\mathbf{K_2Cp^{Sn}}$	$\mathbf{1}_{Sn}$
CCDC ref. code	2386821	2386818	2386820
Empirical formula	$C_{48}H_{72}ErKN_2O_6Si_3$	$C_{20}H_{40}K_2O_2Si_2Sn$	$C_{38}H_{68}ErKN_2O_6Si_2Sn$
Formula weight (g/mol)	1062.03	565.59	1030.17
Temperature/K	100.00	100.00	100.00
Crystal system	monoclinic	monoclinic	monoclinic
Space group	$P2_1/n$	$P2_1/c$	$P2_1/c$
a/Å	15.0638(9)	20.8793(11)	13.2174(5)
b/Å	20.3697(12)	20.8140(12)	16.7130(7)
c/Å	16.8197(10)	13.5530(7)	20.4611(8)
α/°	90	90	90
β/°	90.361(3)	105.607(2)	100.9465(13)
γ/°	90	90	90
V/Å^3	5160.9(5)	5672.7(5)	4437.7(3)
Z	4	8	4
ρ_{calc}/g cm^{-3}	1.367	1.324	1.542
μ/mm^{-1}	6.438	7.255	10.322
F(000)	2201.0	2336.0	2092.0
Crystal size/mm^3	0.15 × 0.1 × 0.05	0.08 × 0.06 × 0.04	0.07 × 0.05 × 0.03
2θ range for data collection/°	6.832 to 107.812	3.822 to 121.432	5.926 to 108.048
Index ranges	-18 ≤ h ≤ 18, -24 ≤ k ≤ 24, -20 ≤ l ≤ 20	-27 ≤ h ≤ 26, -27 ≤ k ≤ 26, -17 ≤ l ≤ 17	-15 ≤ h ≤ 15, -20 ≤ k ≤ 20, -24 ≤ l ≤ 24
Reflections collected	66595	78588	174167
Independent reflections	9402 [R_{int} = 0.0495, R_{sigma} = 0.0406]	12948 [R_{int} = 0.0779, R_{sigma} = 0.0630]	8108 [R_{int} = 0.0393, R_{sigma} = 0.0191]
Data/restraints/parameters	9402/200/557	12948/89/503	8108/0/468
Goodness-of-fit on F^2	1.070	1.140	1.151
Final *R* indexes [I>=2σ (I)]	R_1 = 0.0887 wR_2 = 0.2251	R_1 = 0.0715 wR_2 = 0.1975	R_1 = 0.0314 wR_2 = 0.0820
Final *R* indexes [all data]	R_1 = 0.0921 wR_2 = 0.2270	R_1 = 0.0925 wR_2 = 0.2174	R_1 = 0.0314 wR_2 = 0.0821
Largest diff. peak/hole/e Å^{-3}	2.56/-1.93	1.38/-1.56	2.33/-1.19

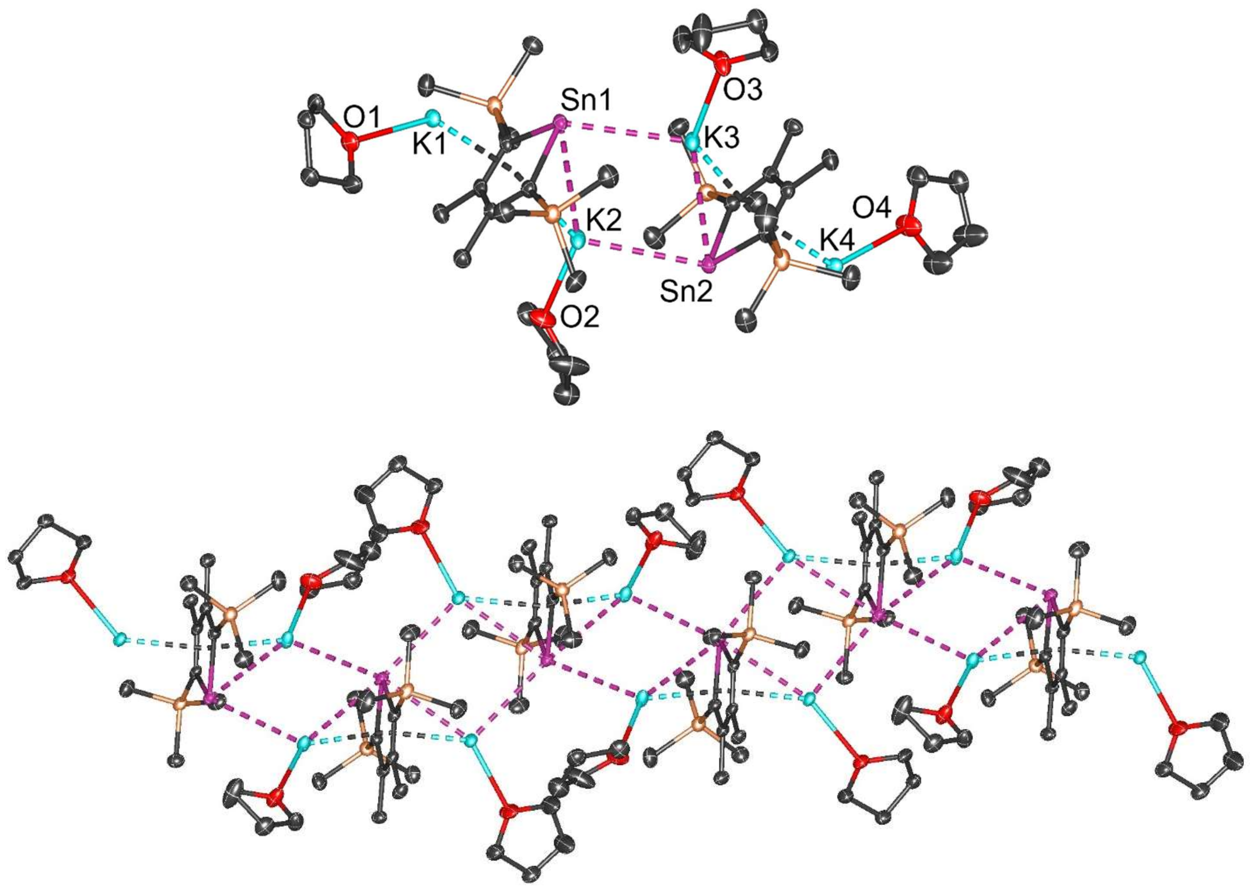

Figure S8. Molecular structures of $K_2Cp^{Sn}{\cdot}2(THF)$. Upper: asymmetric unit. Lower: extended polymeric structure. Thermal ellipsoids are set to 50% probability. Unlabelled atoms in black are carbon. For clarity, hydrogen atoms are not shown.

Table S2. Selected distances and angles for $K_2Cp^{Sn}{\cdot}2(THF)$.

	**Length/Å**	**Angle/°**
K1–C1	3.094(6)	
K1–C2	2.883(5)	
K1–C3	2.916(5)	
K1–C4	3.151(5)	
K1–Sn1	3.5129(13)	
K1–Sn2	3.5224(12)	
K1–O1	2.663(5)	
K2–C1	3.071(5)	
K2–C2	2.908(5)	
K2–C3	2.973(5)	
K2–C4	3.174(5)	
K2–Sn1	3.4743(12)	
K2–O2	2.659(5)	
C1–Sn1	2.158(6)	
C4–Sn1	2.133(5)	

C1–C2	1.415(8)	
C2–C3	1.424(8)	
C3–C4	1.421(8)	
K1–Cp^{Sn} centroid	2.762(2)	
K2–Cp^{Sn} centroid	2.770(2)	
K1– Cp^{Sn}–K2	164.39(11)	
K3–C13	3.157(5)	
K3–C14	2.957(5)	
K3–C15	2.924(5)	
K3–C16	3.069(5)	
K3–Sn2	3.4550(12)	
K3–O3	2.676(4)	
K4–C13	3.063(5)	
K4–C14	2.906(5)	
K4–C15	2.931(5)	
K4–C16	3.142(5)	
K4–Sn2	3.4535(13)	
K4-O4	2.613(5)	
K4-Sn1	3.5273(13)	
C13-Si3	1.833(6)	
C16–Si4	1.828(6)	
C13–Sn2	2.152(5)	
C16–Sn2	2.150(5)	
C13–C14	1.418(8)	
C14–C15	1.426(8)	
C15–C16	1.405(8)	
K3–Cp^{Sn} centroid	2.760(2)	
K4–Cp^{Sn} centroid	2.747(2)	
K3–Cp^{Sn}–K4	167.29(11)	

Table S3. Selected distances (Å) and angles (°) for $\mathbf{1_{Si}}$ and $\mathbf{1_{Sn}}$.

	$\mathbf{1_{Si}}$	$\mathbf{1_{Sn}}$
Er1–C1	2.598(8)	2.622(3)
Er1–C2	2.582(7)	2.598(3)
Er1–C3	2.609(7)	2.595(3)
Er1–C4	2.600(8)	2.596(3)
Er1–E	2.882(2)	3.0820(4)
Er–Cp^E centroid	2.279(3)	2.2758(13)
Er1–C13	2.520(13)	2.532(4)
Er1–C14	2.543(13)	2.554(4)
Er1–C15	2.663 (12)	2.560(4)
Er1–C16	2.619(12)	2.547(4)
Er1–C17	2.579(11)	2.524(4)
Er1–C18	2.555(11)	2.529(4)
Er1–C19	2.471(12)	2.534(4)
Er1–C20	2.479(12)	2.533(4)
Er–COT centroid	1.789(4)	1.7653(18)
COT-Er-Cp^E	171.66(15)	176.07(7)

Magnetic Property Measurements

Magnetic measurements were performed using a Quantum Design MPMS3 SQUID magnetometer equipped with a 7 T magnet. Samples were prepared by gently crushing the crystalline materials before transferring them to a 7 mm NMR tube and covering them in eicosane. Then the tubes were flame sealed under a static vacuum. The eicosane was melted in warm water. Diamagnetic corrections were performed using Pascal's constants.[7]

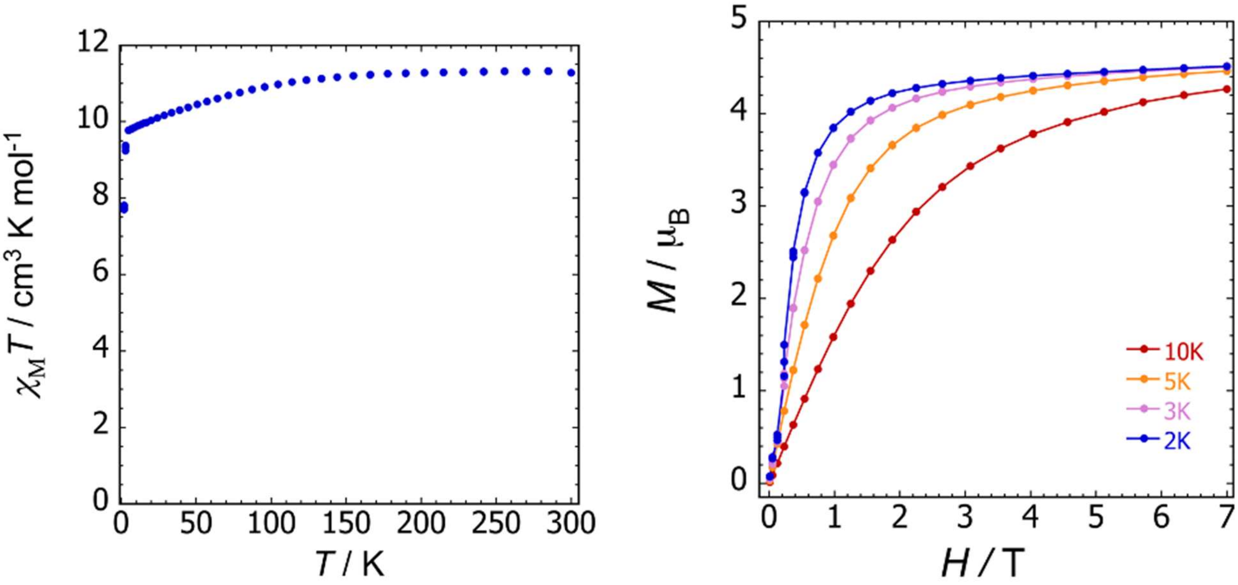

Figure S9. Magnetic susceptibility (left) and isothermal field-dependent magnetization (right) plot for $\mathbf{1}_{Si}$. The value of $\chi_M T$ at 300 K is 11.28 cm^3 K mol^{-1} and the value of M at 2 K and 7 T is 4.51 μ_B.

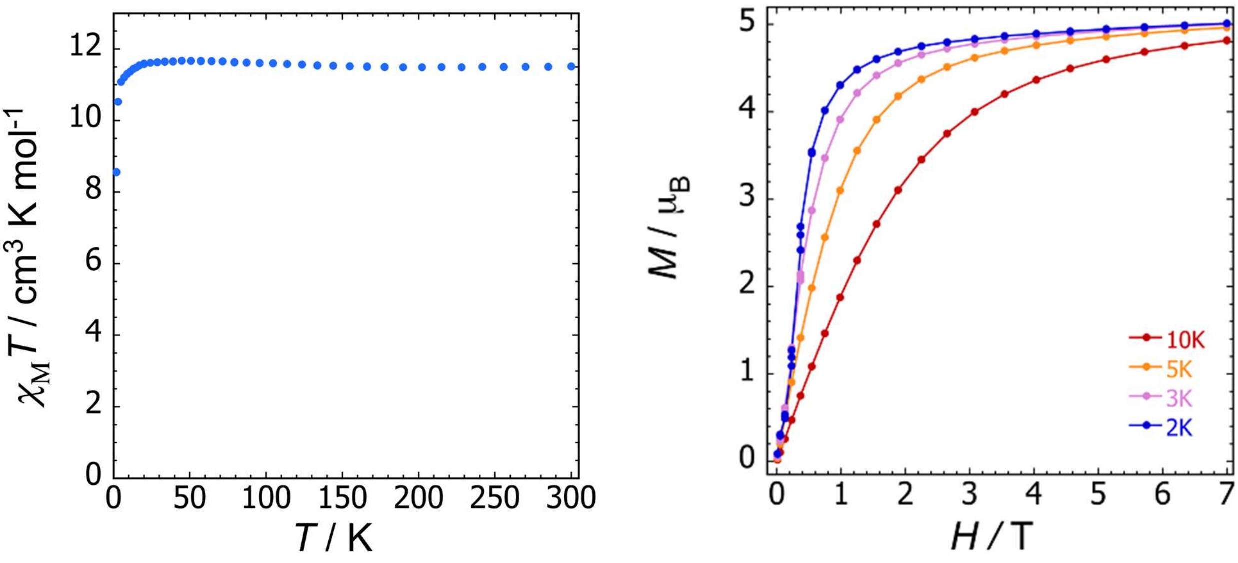

Figure S10. Magnetic susceptibility (left) and isothermal field-dependent magnetization (right) plot for $\mathbf{1}_{Sn}$. The value of $\chi_M T$ at 300 K is 11.50 cm^3 K mol^{-1} and the value of M at 2.0 K and 7 T is 5.00 μ_B.

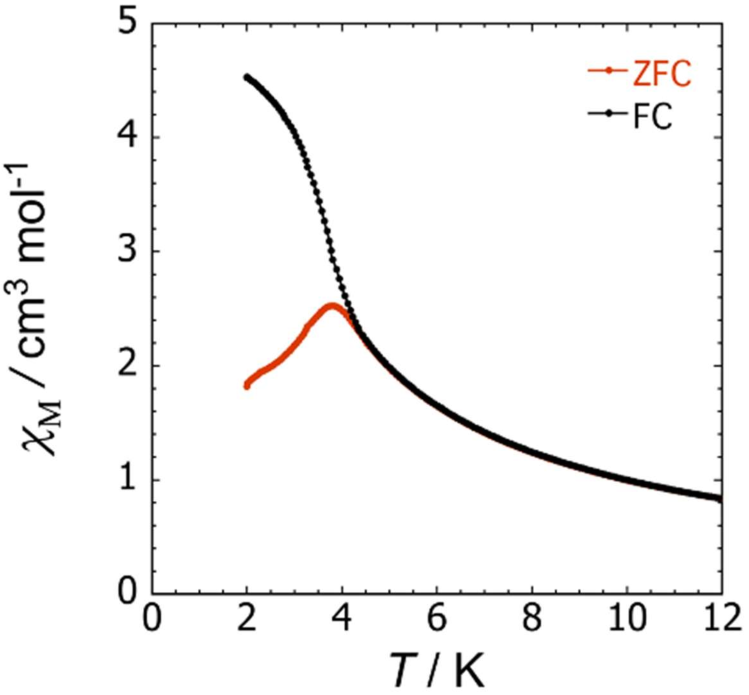

Figure S11. Plot of zero-field cooled and field-cooled (H_{DC} = 1000 Oe) magnetic susceptibility versus temperature for $\mathbf{1}_{\mathbf{Si}}$.

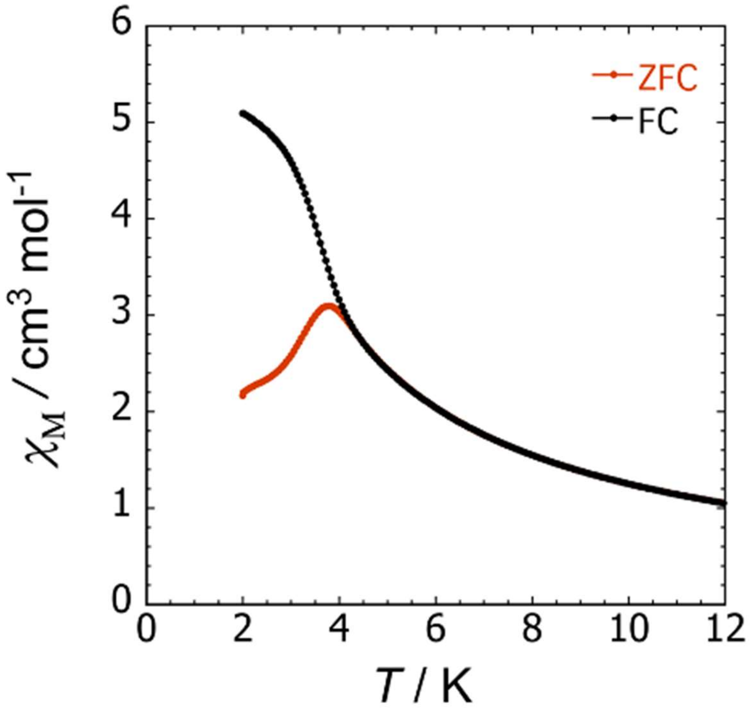

Figure S12. Plot of zero-field cooled and field-cooled (H_{DC} = 1000 Oe) magnetic susceptibility versus temperature for $\mathbf{1}_{\mathbf{Sn}}$.

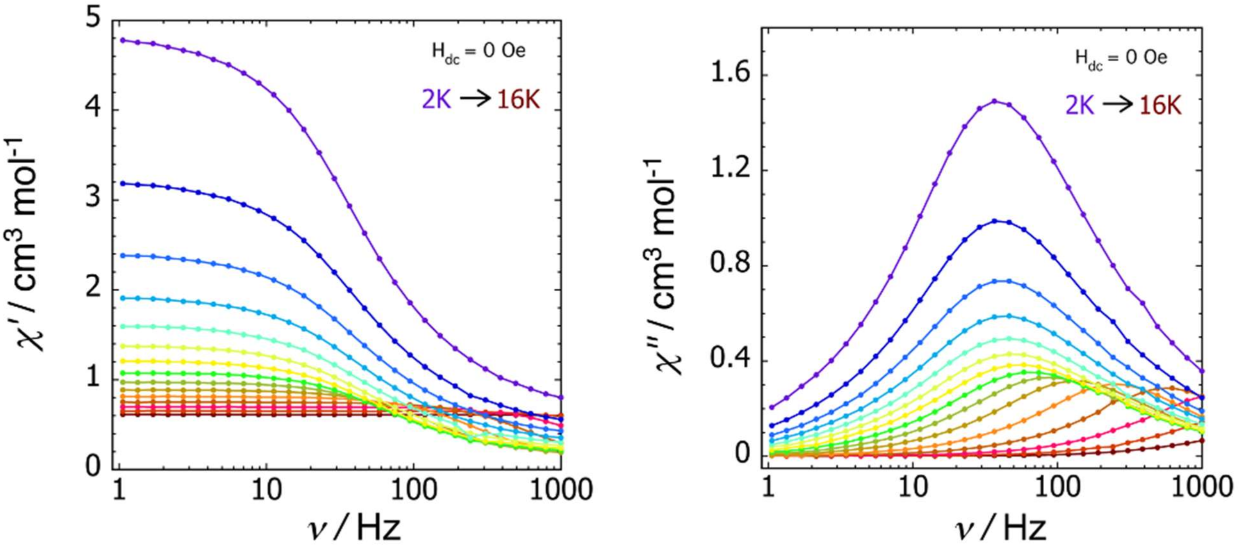

Figure S13. Left: Frequency dependence of the in-phase susceptibility (χ') and right: out-of-phase susceptibility (χ'') for **1$_{Si}$** in zero DC field at ν = 1 to 999 Hz and temperatures of 2-16 K.

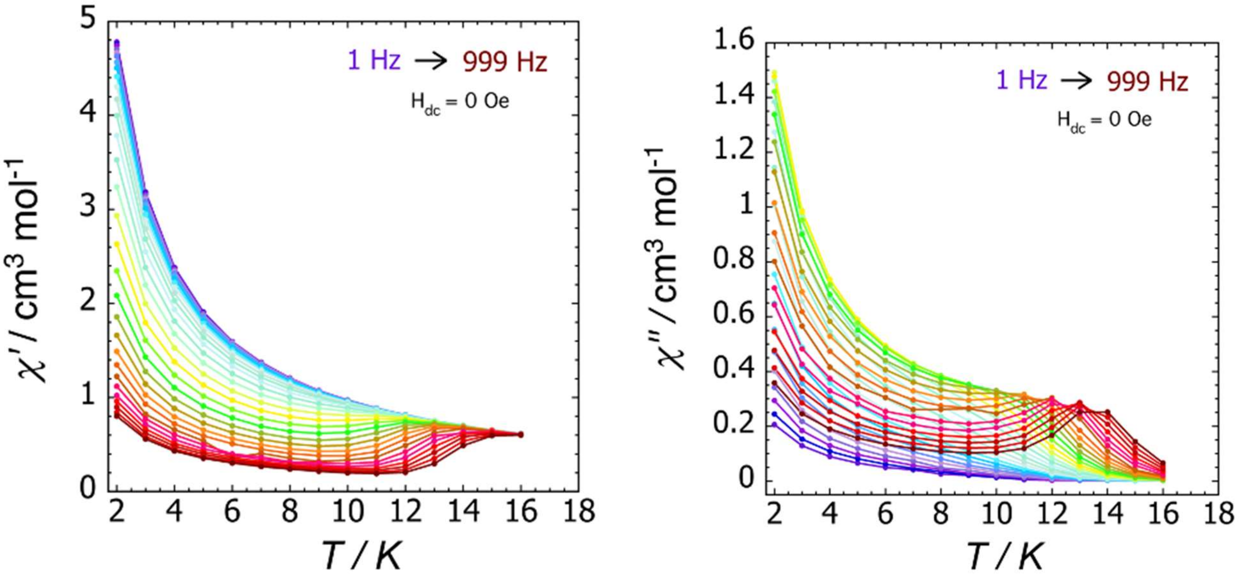

Figure S14. Left: Temperature dependence of the in-phase susceptibility (χ') and right: out-of-phase susceptibility (χ'') for **1$_{Si}$** in zero DC field with ν = 1-999 Hz.

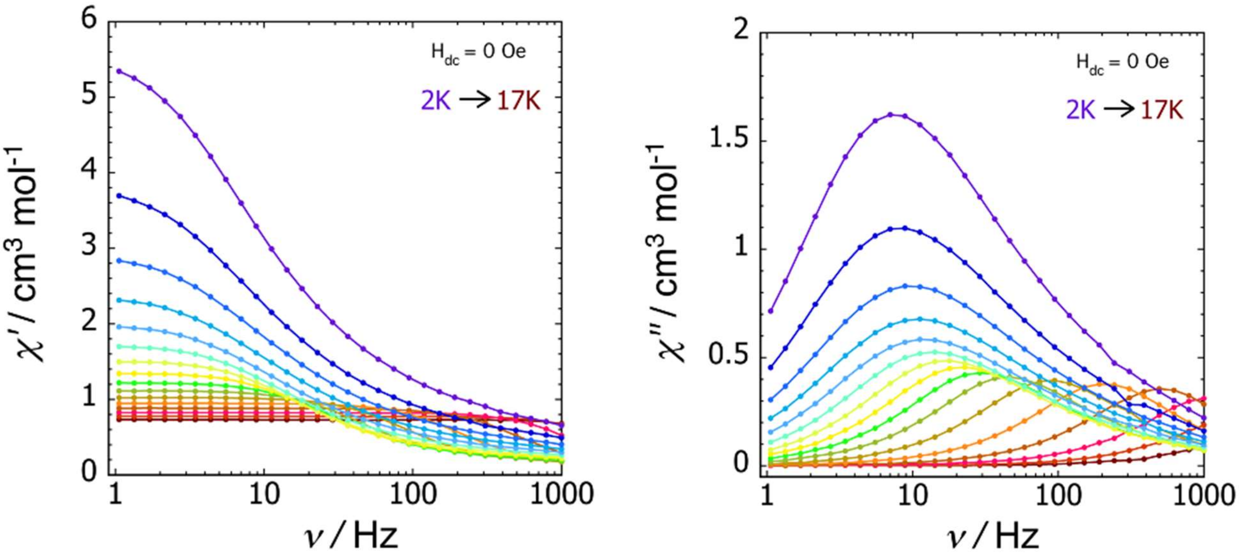

Figure S15. Left: Frequency dependence of the in-phase susceptibility (χ') and right: out-of-phase susceptibility (χ'') for $\mathbf{1}_{\mathbf{Sn}}$ in zero DC field at ν = 1 to 999 Hz and temperatures of 2-17 K.

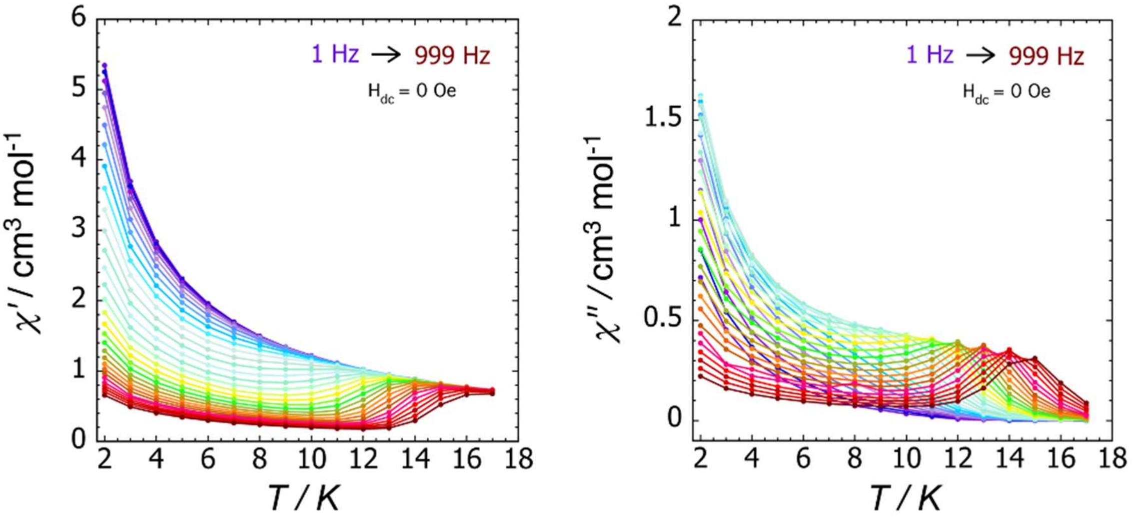

Figure S16. Left: Temperature dependence of the in-phase susceptibility (χ') and right: out-of-phase susceptibility (χ'') for $\mathbf{1}_{\mathbf{Sn}}$ in zero DC field with ν = 1-999 Hz.

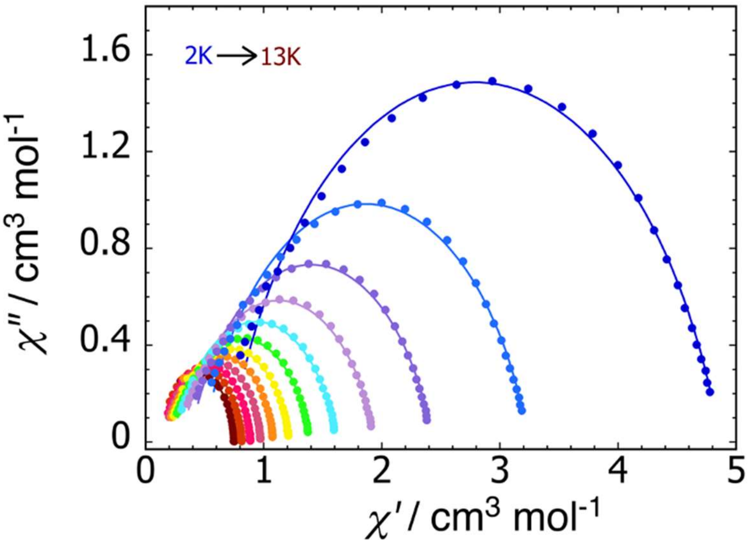

Figure S17. Cole-Cole plots for the AC susceptibilities in zero DC field for $\mathbf{1_{Si}}$ from 2-13 K. Solid lines represent fits to the data using equations 1 and 2.

$$\chi'(\nu_{ac}) = \chi_\infty + \frac{(\chi_s - \chi_\infty)[1+(2\pi\nu_{ac}\tau)^{1-\alpha}\sin(\alpha\pi/2)]}{1+2(2\pi\nu_{ac}\tau)^{1-\alpha}\sin(\alpha\pi/2)+(2\pi\nu_{ac}\tau)^{2(1-\alpha)}} \quad \text{Equation 1}$$

$$\chi''(\nu_{ac}) = \frac{(\chi_s - \chi_\infty)(2\pi\nu_{ac}\tau)^{1-\alpha}\cos(\alpha\pi/2)}{1+2(2\pi\nu_{ac}\tau)^{1-\alpha}\sin(\alpha\pi/2)+(2\pi\nu_{ac}\tau)^{2(1-\alpha)}} \quad \text{Equation 2}$$

Table S4. Relaxation fitting parameters for $\mathbf{1_{Si}}$ corresponding to Figure S16.

***T*/K**	**χ_S/cm^{3}mol^{-1})**	**χ_T/cm^{3}mol^{-1})**	α	**τ/s**
2.000152	0.70851	4.8652	0.20954	0.0037648
3.000392	0.48601	3.2430	0.21074	0.0036064
3.999800	0.37518	2.4310	0.21157	0.0034475
4.999653	0.30539	1.9460	0.21145	0.0032783
6.000968	0.24792	1.6265	0.20496	0.0030676
6.999992	0.23058	1.3991	0.19564	0.0029133
7.999982	0.20886	1.2242	0.17294	0.0026384
8.995888	0.18534	1.0881	0.15311	0.0022174
9.996844	0.17206	0.97919	0.12607	0.0017418
11.000078	0.15649	0.88880	0.093976	0.0011705
11.999729	0.13955	0.81303	0.068375	0.00062327
12.999760	0.12154	0.75094	0.060522	0.00027792

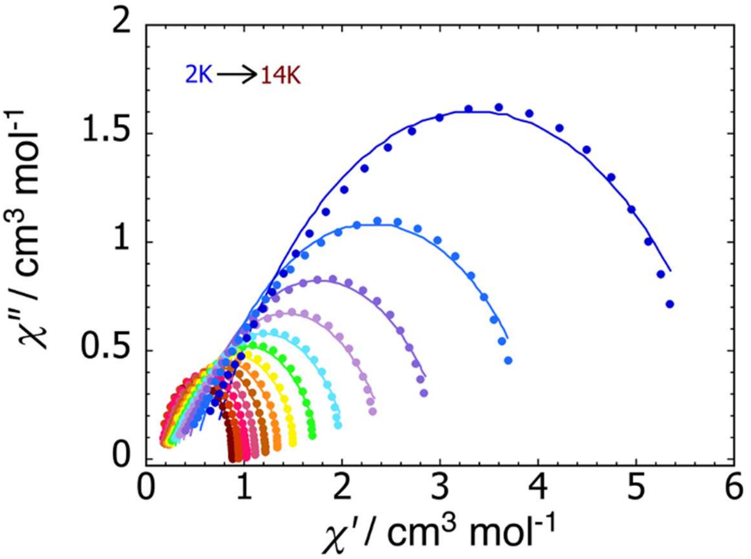

Figure S18. Cole-Cole plots for the AC susceptibilities in zero DC field for $\mathbf{1_{Sn}}$ from 2-14 K. Solid lines represent fits to the data using equations 1 and 2.

Table S5. Relaxation fitting parameters for $\mathbf{1_{Sn}}$ corresponding to Figure S17.

***T*/K**	**χ_S/cm^{3}mol^{-1}**	**χ_T/cm^{3}mol^{-1}**	α	**τ/s**
2.0000	0.63896	6.0535	0.31945	0.017771
3.0000	0.47260	4.1408	0.31988	0.016036
3.9999	0.38639	3.1333	0.31306	0.013616
4.9999	0.33196	2.5225	0.30002	0.012007
5.9997	0.29597	2.1080	0.27634	0.010716
6.9999	0.27203	1.7998	0.23931	0.0092536
7.9996	0.24855	1.5672	0.20088	0.0076460
8.9999	0.22996	1.3849	0.15874	0.0061412
9.9998	0.21034	1.2434	0.12331	0.0046935
11.000	0.19032	1.1267	0.088279	0.0031759
12.000	0.16774	1.0293	0.055186	0.0017025
13.000	0.15263	0.94860	0.029167	0.00075786
14.000	0.15365	0.88154	0.010864	0.00031917

Computational Details

All calculations were carried out on the coordinates obtained from the X-ray structures using ORCA 5.0.2.[62] Hydrogen atom positions were optimized at the DFT level using a pure GGA PBE exchange correlation functional,[63,64] keeping the position of other atoms constant. The calculations were of the CASSCF/QDPT/SINGLE_ANISO type, and the DKH (Douglas-Kroll-Hess) Hamiltonian was used throughout to account for relativistic effects. The SARC2-DKH-QZVP basis set was used for Er(III), SARC-DKH-TZVP for Sn, DKH-def2-TZVP for Si, and all other atoms were treated with the DKH-def2-SVP basis set in combination with the 'AutoAux' auxiliary basis set.[66,67,69] In the active space, we considered 11 electrons in 7 f-orbital, i.e., CAS (7,11) for all complexes. Furthermore, 35 quartet and 90 doublet states were solved in the state-averaged (SA) calculations. To consider spin-orbit coupling, we used the quasi-degenerate perturbation theory (QDPT) approach using SA-CASSCF wave functions.[68] The SINGLE_ANISO module[65] in ORCA was used to compute the g-tensors and crystal field parameters of the low-lying excited state using previously calculated spin-orbit states.

Table S6. SINGLE_ANISO computed crystal-field parameters for $\mathbf{1}_{Si}$ and $\mathbf{1}_{Sn}$.

k	Q	$\mathbf{1}_{Si}$	$\mathbf{1}_{Sn}$
	-2	0.2572E+00	-0.3946E+00
	-1	0.1094E+01	-0.4289E+00
2	0	-0.3059E+00	-0.4879E+00
	1	0.1075E+01	0.7046E-01
	2	0.3282E+00	0.1093E+00
	-4	-0.3730E-03	0.6974E-03
	-3	0.6860E-02	-0.4182E-02
	-2	0.1596E-02	-0.3698E-02
	-1	0.1121E-02	0.7449E-03
4	0	-0.5443E-02	-0.5497E-02
	1	0.1136E-02	0.5025E-02
	2	0.2596E-02	0.6104E-03
	3	-0.1346E-01	0.7482E-02
	4	-0.4811E-03	0.1241E-02
	-6	0.2659E-04	-0.1001E-04
	-5	0.2505E-03	0.3109E-03
	-4	-0.4286E-04	0.2067E-04
	-3	0.1749E-03	-0.1012E-03
	-2	-0.8103E-04	-0.5598E-05
	-1	-0.1589E-03	0.7781E-04
6	0	-0.2349E-04	-0.1839E-04
	1	-0.2265E-03	-0.5434E-04
	2	-0.4406E-05	0.1536E-04
	3	-0.3277E-03	0.1863E-03
	4	-0.2410E-04	0.4407E-04
	5	0.2784E-03	0.3897E-04
	6	0.5706E-05	0.1077E-04

Table S7. Ab initio calculated energy (cm^{-1}) of the low-lying spin-orbit states for $\mathbf{1_{Si}}$ and $\mathbf{1_{Sn}}$.

$\mathbf{1_{Si}}$	$\mathbf{1_{Sn}}$
0.000	0.000
0.000	0.000
104.8311	135.3104
104.8311	135.3104
121.6448	141.9846
121.6448	141.9846
137.1120	159.6340
137.1120	159.6340
167.4583	185.8315
167.4583	185.8315
179.9344	202.6289
179.9344	202.6289
205.0026	215.2829
205.0026	215.2829
302.6141	253.1774
302.6141	253.1774

Table S8. Energies of the KDs, g-tensors and wavefuction composition for **1_{Si}**.

<table>
<tr><th>KD</th><th>E/cm^{-1}</th><th>g_x</th><th>g_y</th><th>g_z</th><th>Wavefunction composition</th></tr>
<tr><td>1</td><td>0.000</td><td>0.0075</td><td>0.0114</td><td>17.52</td><td>94.1%|±15/2>+4.30%|±9/2>+0.90%|±11/2></td></tr>
<tr><td>2</td><td>104.831</td><td>1.552</td><td>2.749</td><td>13.31</td><td>26.20%|±1/2>+32.50%|±3/2>+9.80%|±5/2>+17.60%|±7/2>+5.10%|±9/2>+3.0%|±11/2>+5.50%|±13/2></td></tr>
<tr><td>3</td><td>121.645</td><td>0.842</td><td>3.438</td><td>9.486</td><td>22.40%|±1/2>+21.50%|±3/2>+20.0%|±5/2>+15.60%|±7/2>+7.90%|±9/2>+4.30%|±11/2>+7.40%|±13/2>+1.0%|±15/2></td></tr>
<tr><td>4</td><td>137.112</td><td>3.456</td><td>5.036</td><td>9.606</td><td>29.80%|±1/2>+10.0%|±3/2>+39.80%|±5/2>+4.80%|±7/2>+4.80%|±9/2>+2.80%|±11/2>+7.80%|±13/2></td></tr>
<tr><td>5</td><td>167.458</td><td>1.871</td><td>3.069</td><td>10.14</td><td>12.30%|±1/2>+25.10%|±3/2>+16.0%|±5/2>+12.10%|±7/2>+10.40%|±9/2>+15.0%|±11/2>+8.10%|±13/2>+1.10%|±15/2></td></tr>
<tr><td>6</td><td>179.934</td><td>2.062</td><td>4.003</td><td>6.855</td><td>6.20%|±1/2>+10.10%|±3/2>+7.10%|±5/2>+38.20%|±7/2>+5.70%|±9/2>+25.70%|±11/2>+5.90%|±13/2>+1.0%|±15/2></td></tr>
<tr><td>7</td><td>205.003</td><td>0.659</td><td>1.490</td><td>12.41</td><td>2.40%|±1/2>+0.80%|±3/2>+5.80%|±5/2>+3.90%|±7/2>+37.20%|±9/2>+0.40%|±11/2>+47.70%|±13/2>+1.80%|±15/2></td></tr>
<tr><td>8</td><td>302.614</td><td>0.0314</td><td>0.0417</td><td>17.101</td><td>0.70%|±1/2>+0.10%|±3/2>+1.0%|±5/2>+8.0%|±7/2>+24.60%|±9/2>+47.90%|±11/2>+17.60%|±13/2></td></tr>
</table>

Table S9. Energies of the KDs, g-tensors and wavefuction composition for **1**$_{\mathbf{Sn}}$.

<table>
<tr><th>KD</th><th>E/cm^{-1}</th><th>g_x</th><th>g_y</th><th>g_z</th><th>Wavefunction composition</th></tr>
<tr><td>1</td><td>0.000</td><td>3.4×10^{-4}</td><td>1.2×10^{-3}</td><td>17.80</td><td>98%|±15/2>+0.50%|±11/2>+1.0%|±9/2></td></tr>
<tr><td>2</td><td>135.310</td><td>1.394</td><td>3.448</td><td>13.64</td><td>22.1%|±1/2>+44.8%|±3/2>+12.7%|±5/2>+8.5%|±7/2>+2.0%|±9/2>+1.8%|±11/2>+7.9%|±13/2></td></tr>
<tr><td>3</td><td>141.985</td><td>0.535</td><td>4.967</td><td>9.75</td><td>56.8%|±1/2>+6.3%|±3/2>+24.3%|±5/2>+5.8%|±7/2>+0.9%|±9/2>+1.4%|±11/2>+4.2%|±13/2></td></tr>
<tr><td>4</td><td>159.634</td><td>6.245</td><td>5.518</td><td>1.833</td><td>8.2%|±1/2>+31.1%|±3/2>+19.3%|±5/2>+19.8%|±7/2>+7.0%|±9/2>+1.0%|±11/2>+13.5%|±13/2></td></tr>
<tr><td>5</td><td>185.832</td><td>1.051</td><td>2.570</td><td>9.55</td><td>8.2%|±1/2>+13.9%|±3/2>+38.60%|±5/2>+10.30%|±7/2>+5.30%|±9/2>+2.20%|±11/2>+21.30%|±13/2>+0.10%|±15/2></td></tr>
<tr><td>6</td><td>202.629</td><td>0.384</td><td>1.844</td><td>12.11</td><td>0.4%|±1/2>+1.20%|±3/2>+1.40%|±5/2>+42.3%|±7/2>+1.50%|±9/2>+11.20%|±11/2>+41.70%|±13/2>+0.30%|±15/2></td></tr>
<tr><td>7</td><td>215.283</td><td>0.476</td><td>0.684</td><td>14.58</td><td>3.30%|±1/2>+2.20%|±3/2>+3.40%|±5/2>+9.50%|±7/2>+57.50%|±9/2>+22.90%|±11/2>+0.90%|±13/2>+0.50%|±15/2></td></tr>
<tr><td>8</td><td>253.177</td><td>0.186</td><td>0.241</td><td>16.34</td><td>0.90%|±1/2>+0.50%|±3/2>+0.10%|±5/2>+3.80%|±7/2>+24.8%|±9/2>+53.60%|±11/2>+10.50%|±13/2>+0.50%|±15/2></td></tr>
</table>

Table S10. Magnitudes of transition magnetic moment matrix elements (in μ_B) for $\mathbf{1_{Si}}$.

Climbing Transition			Crossing Transition		
Initial KD	Final KD	Magnitude	Initial KD	Final KD	Magnitude
1	2	0.812916	1	1	0.003168
1	3	0.71940	1	2	0.1184
1	4	0.674096	1	3	0.40947
1	5	0.43073	1	4	0.07283
1	6	0.48135	1	5	0.44019
1	7	0.9631	1	6	0.26724
1	8	0.69411	1	7	0.054306
2	3	1.8938	1	8	0.002161
2	4	1.3442	2	2	2.0046
2	5	0.81908	2	3	1.93331
2	6	0.26844	2	4	0.56560
2	7	0.52239	2	5	0.85929
2	8	0.2322	2	6	1.02081
3	4	1.7366	2	7	0.25479
3	5	1.28380	2	8	0.34882
3	6	1.07202	3	3	1.7895
3	7	0.76709	3	4	1.9955
3	8	0.56013	3	5	0.79574
4	5	1.61277	3	6	0.67282
4	6	1.40997	3	7	0.3316
4	7	0.5532	3	8	0.39739
4	8	0.62012	4	4	2.4403
5	6	1.1266	4	5	1.2884
5	7	1.0556	4	6	0.38134
5	8	0.2946	4	7	0.34139
6	7	2.3617	4	8	0.17130
6	8	0.99173	5	5	1.9910
7	8	1.5073	5	6	2.4456
			5	7	0.88050
			5	8	0.36204
			6	6	1.0198
			6	7	0.5975
			6	8	0.19748
			7	7	0.38137
			7	8	0.13131
			8	8	0.014161

Table S11. Magnitudes of transition magnetic moment matrix elements (in μ_B) for $\mathbf{1_{Sn}}$.

Climbing Transition			Crossing Transition		
Initial KD	Final KD	Magnitude	Initial KD	Final KD	Magnitude
1	2	0.75784	1	1	0.000262
1	3	0.48246	1	2	0.03157
1	4	0.77660	1	3	0.1522
1	5	0.75320	1	4	0.14893
1	6	0.89908	1	5	0.11618
1	7	0.21660	1	6	0.03763
1	8	0.47158	1	7	0.070694
2	3	1.9915	1	8	0.004434
2	4	1.2810	2	2	1.2568
2	5	0.70928	2	3	1.70311
2	6	0.84389	2	4	0.50204
2	7	0.25089	2	5	0.36129
2	8	0.46737	2	6	0.15851
3	4	2.5182	2	7	0.29605
3	5	0.78556	2	8	0.064927
3	6	0.77017	3	3	2.2904
3	7	0.50134	3	4	1.0247
3	8	0.20889	3	5	0.54054
4	5	2.5589	3	6	0.30889
4	6	1.3490	3	7	0.22315
4	7	0.86412	3	8	0.25768
4	8	0.63623	4	4	1.3743
5	6	2.0786	4	5	1.05677
5	7	1.5272	4	6	0.44366
5	8	1.0620	4	7	0.284481
6	7	1.6465	4	8	0.10581
6	8	1.4299	5	5	0.69888
7	8	1.2612	5	6	0.63540
			5	7	0.43870
			5	8	0.14662
			6	6	0.39656
			6	7	0.30380
			6	8	0.18154
			7	7	0.24457
			7	8	0.30230
			8	8	0.08288

References

[76] O. V. Dolomanov, L. J. Bourhis, R. J. Gildea, J. A. K. Howard, H. Puschmann, *J. Appl. Crystallogr.* **2009**, *42*, 339–341.

[77] L. J. Bourhis, O. V. Dolomanov, R. J. Gildea, J. A. K. Howard, H. Puschmann, *Acta Cryst. A* **2015**, *71*, 59–75.

[78] G. M. Sheldrick, *Acta Cryst. C* **2015**, *71*, 3–8.
